# Evaluation of AXIN1 and AXIN2 as targets of tankyrase inhibition in hepatocellular carcinoma cell lines

**DOI:** 10.1038/s41598-021-87091-4

**Published:** 2021-04-02

**Authors:** Wenhui Wang, Pengyu Liu, Marla Lavrijsen, Shan Li, Ruyi Zhang, Shanshan Li, Wesley S. van de Geer, Harmen J. G. van de Werken, Maikel P. Peppelenbosch, Ron Smits

**Affiliations:** 1 Department of Gastroenterology and Hepatology, Erasmus MC-University Medical Center, Rotterdam, The Netherlands; 2 Department of Pharmacology, China Pharmaceutical University, Nanjing, 211198 China; 3 Shenzhen Key Laboratory of Viral Oncology, The Clinical Innovation & Research Centre, Shenzhen Hospital, Southern Medical University, Shenzhen, Guangdong Province China; 4 Department of Urology, Erasmus MC Cancer Institute, Erasmus MC-University Medical Center, Rotterdam, The Netherlands; 5 Cancer Computational Biology Center, Erasmus MC Cancer Institute, Erasmus MC-University Medical Center, Rotterdam, The Netherlands; 6 Department of Gastroenterology and Hepatology, Erasmus MC-University Medical Center, Room Na-1010, Wytemaweg 80, 3015 CN Rotterdam, The Netherlands

**Keywords:** Liver cancer, Cell signalling, Hepatocellular carcinoma, Targeted therapies

## Abstract

AXIN1 mutations are observed in 8–10% of hepatocellular carcinomas (HCCs) and originally were considered to support tumor growth by aberrantly enhancing β-catenin signaling. This view has however been challenged by reports showing neither a clear nuclear β-catenin accumulation nor clearly enhanced expression of β-catenin target genes. Here, using nine HCC lines, we show that AXIN1 mutation or siRNA mediated knockdown contributes to enhanced β-catenin signaling in all AXIN1-mutant and non-mutant lines, also confirmed by reduced signaling in AXIN1-repaired SNU449 cells. Both AXIN1 and AXIN2 work synergistically to control β-catenin signaling. While in the AXIN1-mutant lines, AXIN2 is solely responsible for keeping signaling in check, in the non-mutant lines both AXIN proteins contribute to β-catenin regulation to varying levels. The AXIN proteins have gained substantial interest in cancer research for a second reason. Their activity in the β-catenin destruction complex can be increased by tankyrase inhibitors, which thus may serve as a therapeutic option to reduce the growth of β-catenin-dependent cancers. At concentrations that inhibit tankyrase activity, some lines (e.g. HepG2, SNU398) were clearly affected in colony formation, but in most cases apparently independent from effects on β-catenin signaling. Overall, our analyses show that AXIN1 inactivation leads to enhanced β-catenin signaling in HCC cell lines, questioning the strong statements that have been made in this regard. Enhancing AXIN activity by tankyrase monotherapy provides however no effective treatment to affect their growth exclusively through reducing β-catenin signaling.

## Introduction

Hepatocellular carcinoma (HCC) is a prevalent cancer with worldwide around 700,000 new cases diagnosed yearly, and a leading cause of cancer related deaths^1,2^. Aberrant activation of Wnt/β-catenin signaling is often observed in HCC^3^. Intracellular β-catenin levels are regulated by a multiprotein complex composed of the APC tumor suppressor, scaffold proteins AXIN1, AXIN2 and the kinases GSK3 and CK1α^3,4^. When cells are not exposed to extracellular Wnt ligands, β-catenin is constitutively phosphorylated and degraded to maintain cytoplasmic levels at a minimum. Following Wnt stimulation, the multiprotein complex dissociates resulting in the accumulation of cytosolic and nuclear β-catenin, which in turn triggers the transcription of specific target genes. Aberrant activation of Wnt/β-catenin signaling in HCC has been mainly attributed to activating somatic mutations in the *CTNNB1* gene coding for β-catenin (20–25%)^3,5–8^, for which it is well-accepted that they support tumor growth by enhancing β-catenin signaling in a dominant fashion.

Another component of the Wnt/β-catenin signaling pathway regularly inactivated in HCC is *AXIN1* (8–10%)^3,5,6,9^. Originally, given its role in the β-catenin destruction complex, mutational inactivation of AXIN1 was considered to support HCC development by aberrantly enhancing β-catenin signaling. This view has however been challenged by several reports showing neither a clear nuclear β-catenin accumulation nor clearly enhanced expression of β-catenin target genes in *AXIN1*-mutant HCCs^10–12^. Others have provided some evidence of increased β-catenin signaling in *AXIN1* mutant HCC cells, albeit modest^11,13,14^. Qiao and coworkers showed that HCC induction following AXIN1 deletion in mouse livers was strongly dependent on functional β-catenin, but as argued by others only in the context of simultaneous MET activation^15–17^. Hence, whether β-catenin signaling is activated following AXIN1 mutation and its relevance for supporting human HCC growth is still heavily debated.

The AXIN proteins have gained substantial interest in cancer research for a second reason. Their activity in the β-catenin destruction complex can be increased by tankyrase inhibitors, which thus may serve as a therapeutic option to treat β-catenin-dependent cancers. The AXIN proteins, like β-catenin itself, are under tight proteolytic control. Poly-ADP-ribosyltransferases tankyrase-1 and -2 (encoded by *TNKS/TNKS2*) associate with AXIN, resulting in their PARsylation and subsequent ubiquitylation and degradation, thereby limiting the activity of the destruction complex^18–21^. Blocking tankyrases results in the formation of so-called degradasomes in which all components of the β-catenin destruction complex aggregate, leading to an efficient β-catenin turnover. Application of these tankyrase inhibitors has been investigated for the treatment of various cancer types, with some successful initial results for a subset of tumors^22–30^.

Here, we employed *CTNNB1-*, *AXIN1-* and non-mutant HCC cell lines to investigate the impact of tankyrase inhibition on Wnt/β-catenin signaling as well as growth, and to further explore the function of AXIN1/AXIN2 in regulating Wnt/β-catenin signaling in HCC cells.

## Results

### Baseline levels of AXIN1 and AXIN2 in HCC cell lines

**Figure 1:**
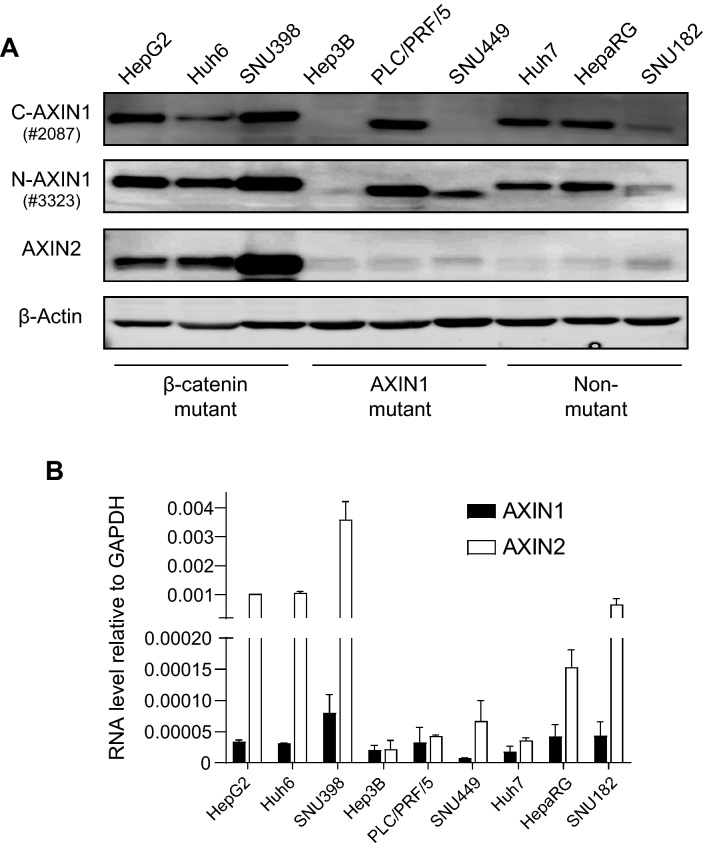
Baseline levels of AXIN1 and AXIN2 in HCC cell lines. (**A**) Western blotting assay showing the basal protein levels of AXIN1 and AXIN2. For AXIN1 both N-terminal and C-terminal antibodies were used, respectively, #3323 and #2087 from Cell Signaling Technology. (**B**) RNA levels tested by TaqMan qRT-PCR (mean ± SD, n = 2, twice). All expression levels are depicted relative to the housekeeping gene *GAPDH*. Note the interrupted Y-axis scale.

To explore the function of AXIN1 and AXIN2 in regulating Wnt/β-catenin signaling in HCC cells, we employed 9 HCC cell lines listed in Supplemental Table S1, in which gene mutations related to Wnt/β-catenin signaling are depicted. By western blotting, AXIN1 was detectable in all lines except Hep3B carrying a homozygous p.R146* mutation (Fig. 1A). AXIN2 was clearly detectable in the β-catenin mutant lines, and weakly visible in the others. Next, we compared *AXIN1* and *AXIN2* RNA expression levels by TaqMan qRT-PCR (Fig. 1B). *AXIN2* was expressed at higher levels than *AXIN1* in most HCC cell lines, independent of their β-catenin related mutation status. In accordance with *AXIN2* being a β-catenin target gene, the expression differences were largest in the CTNNB1 mutant lines.

### Both AXIN1 and AXIN2 contribute to β-catenin signaling regulation in HCC cell lines

To evaluate the functionality of either AXIN1 or AXIN2 to the regulation of β-catenin signaling in each cell line, we silenced expression of each gene separately or in combination, using SMARTpool siRNA mediated knockdown (Supplemental Fig. S1). *APC* knockdown was used as a positive control for activation of β-catenin signaling.

**Figure 2:**
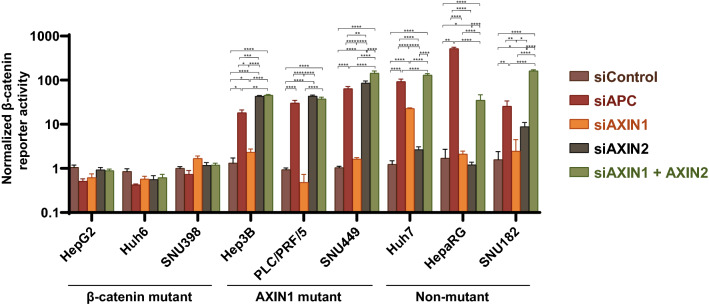
Both AXIN1 and AXIN2 contribute to β-catenin signaling regulation in HCC cell lines. All cell lines were subjected to a β-catenin reporter assay after siRNA-mediated knockdown of *AXIN1*, *AXIN2*, a combination thereof or *APC*. No significant changes in β-catenin reporter activity were observed in the CTNNB1-mutant lines. Among the *AXIN1-*mutant cells (Hep3B, PLC/PRF/5 and SNU449) both *APC* and *AXIN2* knockdown are equally effective in enhancing signaling. *AXIN1* or *AXIN2* knockdown in the non-mutant lines (Huh7, HepaRG, SNU182) results in an incomplete increase in reporter activity when compared with *APC* knockdown, while combined *AXIN1/AXIN2* knockdown is similarly effective. For this experiment the WRE/CMV-Renilla ratio for the control siRNA sample-1 was arbitrarily set to 1 for each cell line, after which all β-catenin reporter WRE/CMV-Renilla ratios for the samples were normalized to this control. Only significant differences are shown using an unpaired t-test (mean ± SD, n = 3, *P < 0.05; **P < 0.01; ***P < 0.001; ****P < 0.0001). Note the logarithmic Y-axis scale. The result shown here is one representative result, but comparable results were observed in two independent experiments performed on relevant lines (Supplemental Fig. S2).

In line with the supposed dominant activity of mutant β-catenin, no significant changes in β-catenin reporter activity were observed in the CTNNB1-mutant lines (Fig. 2, Supplemental Fig. S2). As expected, *APC* knockdown resulted in a strong increase of β-catenin reporter activity in all other lines. Importantly, in all three *AXIN1*-mutant lines a comparable increase in reporter activity was observed as a consequence of solely *AXIN2* knockdown. *AXIN1* knockdown in these lines showed however no significant effect on reporter activity. These results show that AXIN2 is expressed at biologically functional levels in the *AXIN1*-mutant lines and confirm that the *AXIN1* mutation impairs its role in β-catenin regulation.

Among the non-mutant cell lines a variable response was noted upon *AXIN1* or *AXIN2* knockdown. In Huh7 cells knockdown of only *AXIN1* led to a strong increase in reporter activity, while this was the case for *AXIN2* in SNU182. HepaRG cells were barely affected in β-catenin reporter activity with knockdown of either gene separately. However, simultaneous knockdown of *AXIN1* and *AXIN2* led to a robust induction of β-catenin reporter activity in all non-mutant lines.

We also performed qRT-PCR analyses for the β-catenin target gene *AXIN2*. *APC* knockdown increased *AXIN2* expression in all AXIN1- and non-mutant lines, while no change was observed in the CTNNB1-mutant line SNU398 used as control (Supplemental Fig. S3). *AXIN1* knockdown led to a 2–4-fold increase in *AXIN2* levels in all non-mutant lines, while as expected no clear change was observed in the AXIN1-mutant lines. A meaningful interpretation of *AXIN2* knockdown is strongly complicated by the fact that total *AXIN2* RNA levels are simultaneously downregulated by siRNA as well as upregulated by enhanced β-catenin signaling (a more detailed explanation is provided accompanying Supplemental Fig. S1). Hence, for the interpretation of the *AXIN2* knockdown experiments we restricted ourselves to the reporter assays.

Taken together, these analyses show that (i) in HCC cell lines both AXIN1 and AXIN2 work synergistically to control β-catenin signaling; (ii) in the AXIN1-mutant lines, AXIN2 is solely responsible for keeping signaling in check, whereas in the non-mutant lines both AXIN proteins contribute to β-catenin regulation to varying levels depending on the cell line under investigation; (iii) in the non-mutant lines *AXIN1* knockdown leads to an increase in β-catenin reporter activity and/or *AXIN2* expression.

### Effects on β-catenin signaling following tankyrase inhibition

**Figure 3:**
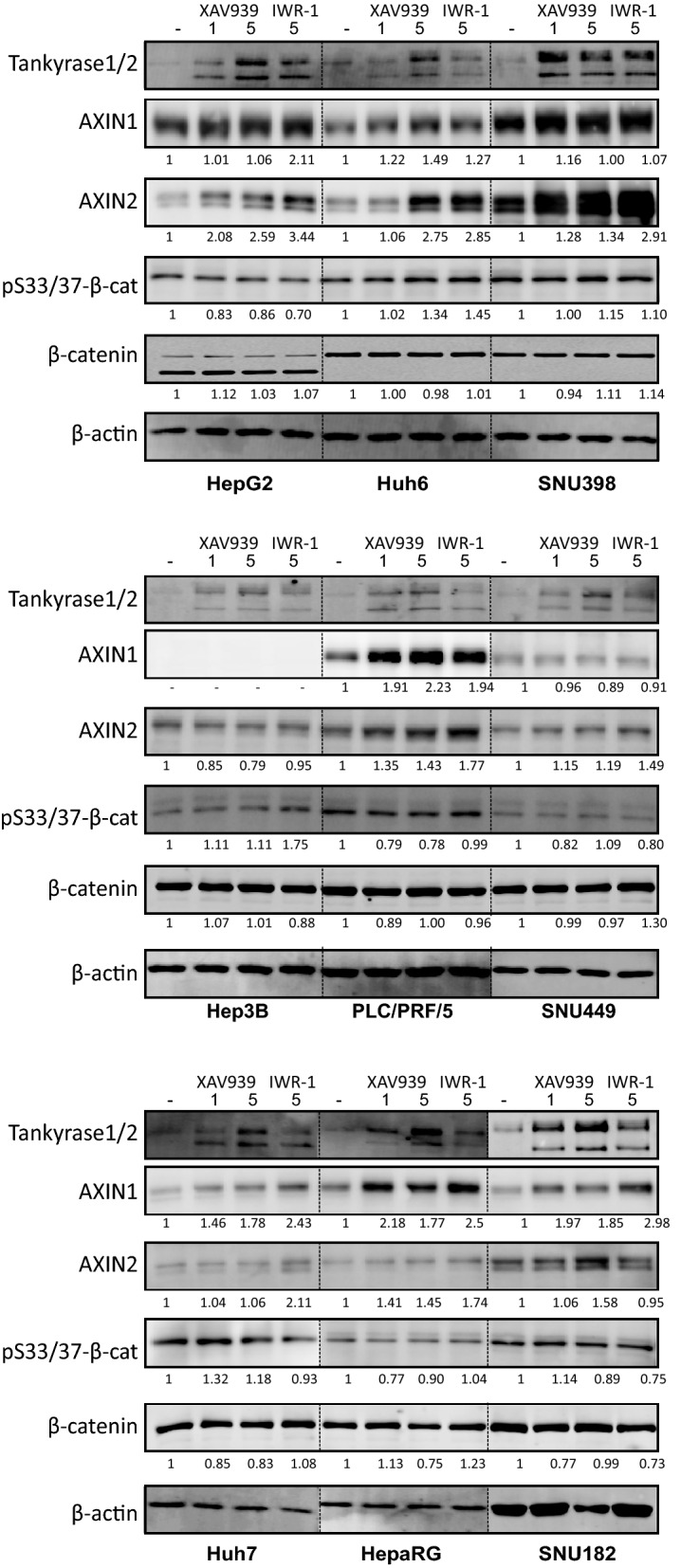
Effect of tankyrase inhibition on indicated proteins. Western blotting assay showing levels of tankyrase-1/2, AXIN1/2, p33/37-β-catenin (pS33/37-β-cat) and total β-catenin after 1-5 μM XAV939 or 5 μM IWR-1 for 24 h. Tankyrase-1/2 efficiently accumulate in all cell lines, while variable responses are observed for AXIN1 and AXIN2. No clear change is observed in pS33/37-β-catenin except for Huh6 and Hep3B, in which a modest increase is seen. Total β-catenin levels are not clearly altered.

The tankyrase enzymes have been shown to antagonize the activity of the β-catenin destruction complex by PARsylation and subsequent breakdown of AXINs^18–21^. Their inhibition can lead to an enhanced β-catenin turnover and growth suppression of a subset of β-catenin-dependent cancers^22–30^. All HCC cell lines showed readily detectable expression of TNKS/TNKS2 (Supplemental Fig. S4). We treated HCC cell lines with tankyrase inhibitors XAV939 or IWR-1, using the CRC cell line SW480 as positive control. In accordance with previous studies on SW480 cells, XAV939 and IWR-1 stabilized tankyrase-1/2, AXIN1 and AXIN2, increased pS33/37-β-catenin and diminished total β-catenin levels (Supplemental Fig. S5). Among the HCC cell lines, at least one of the AXIN proteins showed a clear accumulation, with the exception of Hep3B (Fig. 3, Supplemental Fig. S6). In the CTNNB1 mutant lines, AXIN2 was most prominently stabilized, while this was the case for AXIN1 in the non-mutant lines. With respect to β-catenin, a slight increase in pS33/37 phosphorylation was observed only in Huh6 and Hep3B, the latter restricted to IWR-1. Total β-catenin levels were not clearly altered in any of the lines.

In various cell lines tankyrase inhibition has been shown to lead to the formation of so-called β-catenin degradasomes, consisting of higher-order structures in which all components required for β-catenin degradation are present^31^. These degradasomes can be visualized as AXIN- and tankyrase-positive cytoplasmic puncta^32^, which were readily visible in XAV939 treated SW480 cells (Supplemental Fig. S7). This was also the case in Huh6 cells, although not as prominent as in SW480. By western blotting PLC/PRF/5 cells express clear levels of a mutant AXIN1 protein retaining the DIX domain required for multimerization. Accordingly, accumulation of tankyrase- and AXIN1-positive puncta was detectable, but no obvious change in abundance and subcellular localization of AXIN2 was visible. Lastly, in Hep3B cells exclusively tankyrase-positive puncta were observed, in line with the absent/low AXIN1/2 levels observed by western blot.

**Figure 4:**
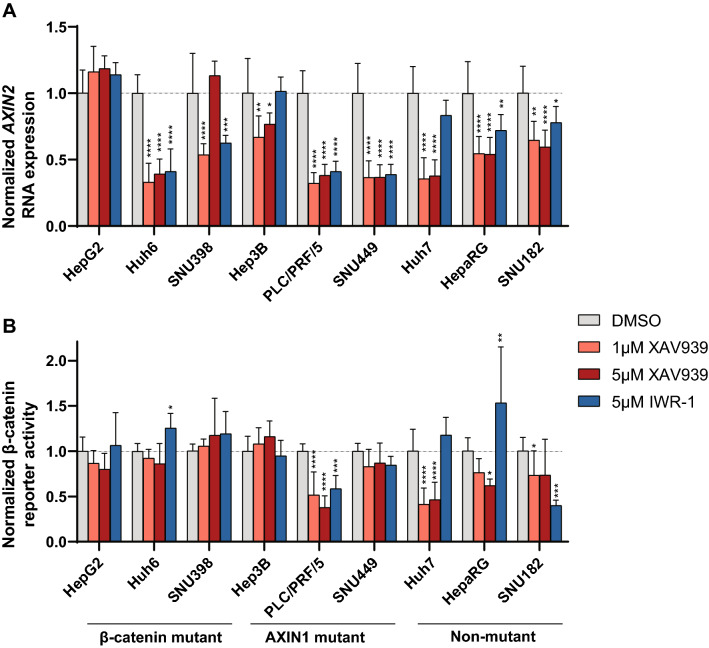
Effect of tankyrase inhibition on Wnt/β-catenin signaling. HCC cell lines were incubated with 1-5 μM XAV939 or 5 μM IWR-1, followed by an *AXIN2* qRT-PCR or a β-catenin reporter assay. (**A**) Fold changes in *AXIN2* expression levels depicted relative to the DMSO control-treated samples, which are arbitrarily set to 1. Only significant changes (unpaired t-test) are shown (mean ± SD, n = 3, twice, *P < 0.05; **P < 0.01; ***P < 0.001; ****P < 0.0001; 24 h treatment). (**B**) WRE/CMV-Renilla β-catenin reporter ratios were obtained for each cell line. Fold changes relative to the DMSO control-treated samples are shown, which are arbitrarily set to 1. Only significant changes (unpaired t-test) are shown (mean ± SD, n = 3, *P < 0.05; **P < 0.01; ***P < 0.001; ****P < 0.0001; 48 h treatment). See also Supplemental Fig. S8 showing absolute *AXIN2* and β-catenin reporter levels and Supplemental Fig. S9.

Combined these data suggest that tankyrase inhibition leads to the stabilization of AXIN1 and/or AXIN2 in most HCC cell lines, while total β-catenin levels are barely affected. To determine the direct consequences on β-catenin signaling following XAV939/IWR-1 treatment, we measured *AXIN2* RNA expression and β-catenin reporter activity (Fig. 4, Supplemental Figs. S8/S9). We observed a 1.2–3-fold reduction in *AXIN2* RNA levels in all lines after treatment with XAV939 or IWR-1, with the exception of HepG2 (XAV939 and IWR-1), and Hep3B and Huh7 (IWR-1). Using the reporter assay, significant reductions with XAV939 were only observed in PLC/PRF/5 and Huh7, whereas this was the case for PLC/PRF/5 and SNU182 using the IWR-1 inhibitor. Importantly, despite the 1.2–3-fold reduction in AXIN2 and/or β-catenin reporter levels observed in some of the lines, the absolute level of nuclear β-catenin signaling remains high in especially the CTNNB1- and AXIN1-mutant lines (Supplemental Fig. S8).

Taken together, these results show that tankyrase inhibition results in some AXIN accumulation in basically all HCC lines tested, while effects on β-catenin accumulation and signaling are non-apparent or modest, respectively.

### Colony formation capability of HCC lines following tankyrase inhibition

**Figure 5:**
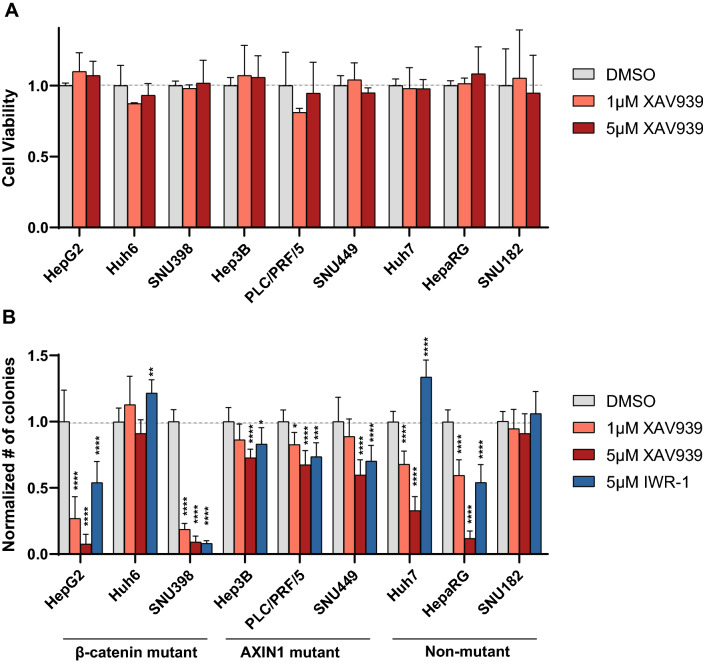
Effect of tankyrase inhibitor XAV939 on the viability and colony formation capacity of HCC lines. (**A**) HCC cell lines were treated with XAV939 at 1 μM or 5 μM for three days followed by a MTT-assay (n = 4). No statistical significant growth reduction was observed (unpaired t-test). (**B**) Fold changes observed in a colony formation assay following XAV939/IWR-1 treatment. Only significant changes are shown (mean ± SD, n = 6, *P < 0.05; **P < 0.01; ***P < 0.001; ****P < 0.0001; unpaired t-test).

Following three days of XAV939 treatment, HCC cell growth was unaltered in a MTT assay, even at higher 5 μM concentration (Fig. 5A). To test the effect of long-term treatment on lower cell numbers, we performed a colony formation assay treating HCC cells with XAV939 or IWR-1 for 2 weeks (Figs. 5B and Fig. S10). Among the β-catenin mutant lines, HepG2 and SNU398 were strongly inhibited in colony formation. As both these lines retained high levels of β-catenin signaling following tankyrase inhibition, this growth reduction is most likely the consequence of other cellular processes regulated by tankyrases, such as telomere maintenance, Hippo signaling, mitosis or DNA strand break repair^30,33–35^, or off-target effects of especially XAV939 on other cellular proteins, such as poly(ADP-ribose) polymerases PARP1 and PARP2^24,36^. Among the AXIN1-mutant cell lines a modest but significant reduction in colony numbers was observed, especially using 5 μM XAV939 and IWR-1. For PLC/PRF/5 this may be attributed to reduced β-catenin signaling, while this is less obvious for Hep3B and SNU449. Lastly, among the non-mutant lines SNU182 was unaffected by tankyrase inhibition. Huh7 was clearly inhibited in colony formation with XAV939, but not using IWR-1, which is in line with the observed effects on β-catenin signaling for both compounds. HepaRG showed reduced numbers, which may be partially explained by reduced β-catenin signaling.

Collectively, these findings suggest that tankyrase inhibition can inhibit the growth of several HCC cell lines when plated at low density. Not in all cases can the growth suppressing effects be linked to β-catenin signaling (e.g. HepG2 and SNU398). Tankyrase inhibition is however less effective when initiated with higher cell concentrations, as shown in the MTT-assay.

Table 1 summarizes all the fold changes that were observed following tankyrase inhibition in western blot, β-catenin signaling and colony formation analyses.

**Table 1:**
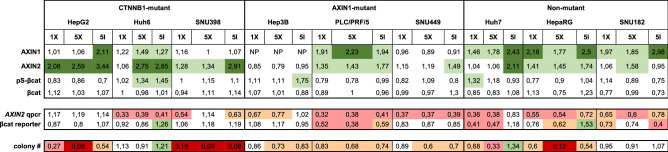
Summary of western blot, β-catenin signaling and colony formation analyses.

Fold changes relative to controls for each analysis are depicted and color-coded. Significant increases are shown in green colors, while decreases are shown in orange/red colors. 1X is 1 μM XAV939; 5X is 5 μM XAV939; 5I is 5 μM IWR-1.

### Restoring full-length AXIN1 expression in SNU449 cells reduces *AXIN2* expression, but has overall minimal effects on cell behavior

**Figure 6:**
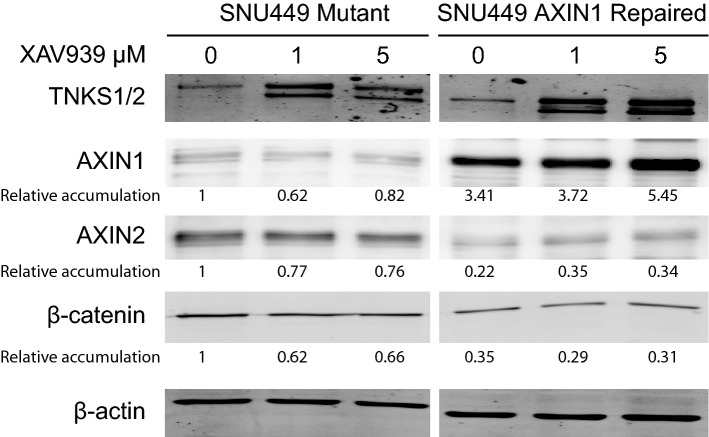
Evaluation of AXIN1-repaired SNU449 cells. At baseline (0 μM XAV939) the AXIN1-repaired SNU449 cells show increased levels of AXIN1, while AXIN2 and total β-catenin levels are decreased, indicating that AXIN1 contributes to increased β-catenin turnover. Following XAV939 treatment at indicated dosages, only a clear accumulation is observed for the AXIN1-repaired protein. For this experiment the AXIN1-repaired SNU449 clone-20 was compared with control clone-2. All relative band intensities are compared to this latter clone, which were arbitrarily set to 1.

To more directly investigate the consequences of AXIN1 mutation for supporting β-catenin signaling and liver cancer growth, we used CRISPR-Cas9 technology to repair the homozygous p.R712* mutation present in SNU449 cells. This truncating mutation removes the C-terminal DIX domain that is essential for AXIN1 to form higher order structures through multimerization and fulfil its function in β-catenin regulation^37,38^. We successfully obtained several independent clones with complete repair of AXIN1 (Supplemental Fig. S11A). Protein expression levels are 2–threefold higher compared to unrepaired clones, which can be attributed to higher expression levels of *AXIN1* RNA (Figs. 6 and Fig. S11B). The latter is most likely resulting from nonsense-mediated decay of the mutant transcript in the parental line. Importantly, all repaired clones show a significant reduction in *AXIN2* expression, in line with an improved β-catenin turnover (Fig. 6 and Supplemental Fig. S11C). When AXIN1-repaired cells are treated with XAV939, they show a clear accumulation of AXIN1, in contrast to unchanged levels of the mutant protein (Fig. 6). Thus, we successfully resulted in restoring AXIN1 expression in SNU449 cells, which was accompanied by reduced expression of the β-catenin target gene *AXIN2*.

The fact that we successfully obtained SNU449 cells with normalized AXIN1 expression also highlights that its mutation is not essential to sustain growth in culture, which was confirmed by comparable cell doubling times in the AXIN1-repaired clones with the parental line (Supplemental Fig. S12). AXIN1 has also been functionally linked to other proteins relevant for tumorigenesis, such as MYC, SMAD3 and TP53^39–42^. To determine in an unbiased manner the consequences of restoring AXIN1 expression in SNU449 cells, we subjected 3 repaired clones and 3 controls to RNA sequencing. Surprisingly, only 5 genes were significantly altered in expression including *AXIN2* (Supplemental Table S2).

In conclusion, the analyses of the AXIN1-repaired SNU449 cells show that in this cell line the *AXIN1* mutation has minimal effects on cell behavior and gene expression. Importantly, reduced expression of the well-established β-catenin target gene *AXIN2* is consistently seen.

## Discussion

Inappropriate activation of Wnt/β-catenin signaling has been reported frequently in HCC. This has been mainly attributed to somatic mutations in the *CTNNB1* gene (20–25%)^3,5–7,9^. A second common mechanism originally considered to lead to enhanced β-catenin signaling, is mutational inactivation of *AXIN1* observed in about 10% of HCCs, putting it among the most frequently mutated genes in HCC^3,5–7,9^. Given its activity in the β-catenin destruction complex this was a logic assumption, however several reports have suggested that AXIN1 mutation leads to liver cancer in the absence of increased β-catenin signaling^10–12^. Recently, HCC formation in mice following mutation of AXIN1 was however shown to strongly dependent on functional β-catenin^15^, but as argued by others only in the context of simultaneous MET activation^16^. Hence, the issue whether *AXIN1* mutation leads to activation of β-catenin signaling is still under debate^16,17^. In addition, the AXIN proteins have gained substantial interest in cancer research because their activity in the β-catenin destruction complex can be increased by tankyrase inhibitors, which thus may serve as a therapeutic option to reduce the growth of β-catenin-dependent cancers. Here, using a panel of 9 HCC cell lines with specific mutations in components of the β-catenin signaling pathway, we have investigated both aspects of AXIN biology.

The conclusion that *AXIN1*-mutant HCCs develop without β-catenin activation is largely based on expression profiling using a 23-β-catenin target gene signature^12^. Most *AXIN1*-mutated HCCs clustered in a group with no evident β-catenin activation program, and only about 20% in a group with weak activation. Interestingly, these same groups also show an enrichment in CTNNB1 mutations within armadillo repeats 5 and 6 (i.e. K335, W383, N387), for which we have recently unequivocally shown that they are efficient inducers of HCC development in mice, associated with a weak but significant activation of β-catenin signaling^8^. Expression profiling of tumor tissues has the inherent shortcoming that average expression is determined of a heterogeneous group of cells, including contaminating normal cells. Small but biologically relevant increases of β-catenin signaling, like the ones imposed by armadillo repeat mutant β-catenin, in a subset of cells can easily be missed. Likewise we feel that it is difficult to use expression profiling to fully exclude increased β-catenin signaling in AXIN1-mutated cancers.

The same holds true for immunohistochemical demonstration of nuclear β-catenin. AXIN1-mutant HCCs predominantly show an exclusive membranous β-catenin staining^10–12,15^, indeed suggesting that there is no nuclear signaling. However, this is also observed in tumors carrying oncogenic CTNNB1 mutations within the armadillo repeats and a subset of S45-mutant cancers^8,43^, while these clearly lead to enhanced signaling. Thus, although nuclear accumulation of β-catenin is a reliable predictor of active signaling, its absence does not exclude that a low level of biologically relevant signaling is active^44^. It shows that both genetic profiling and IHC have their shortcomings to unambiguously determine the absence of nuclear β-catenin signaling.

Our analyses show that all three AXIN1-mutant HCC cell lines have increased β-catenin reporter activity (Supplemental Fig. S8)^14^. Furthermore, we show that *AXIN1* knockdown in three non-mutant lines leads to enhanced signaling, in accordance with other reports^12,15^. In the reverse experiment in which we restore AXIN1 expression in SNU449 cells, we consistently observe reduced expression of the β-catenin target gene *AXIN2*. Taken together, this shows that *AXIN1* mutation leads to a modest increase of β-catenin signaling that may be relevant for hepatocellular tumorigenesis.

In this respect, several examples have been presented in the literature showing that minor alterations in the level of β-catenin signaling can have profound biological effects^4,45–47^. In case of hepatocellular cancer, Buchert et al. have shown that late-onset hepatocellular tumors were present in all mice carrying a hypomorphic *APC* mutation associated with just a modest increase in β-catenin signaling, while tumor formation was absent or largely prevented with slightly increased or decreased signaling^45^. This narrow window of signaling effective in mouse liver cancer formation highlights the importance of low level signaling for some cancer types and shows that it is difficult to fully exclude a role for β-catenin signaling.

AXIN1-mutant tumors depend to a large extent on AXIN2 to counterbalance signals that induce β-catenin signaling, as shown by the strong increase in β-catenin reporter activity that we observe after *AXIN2* knockdown, comparable to levels seen with *APC* siRNA. Hence, they are expected to be more prone to signal in conditions that normally activate the β-catenin signaling pathway. Most liver cancers emerge in patients with chronic liver injury, inflammation and cirrhosis. Under such harsh circumstances, the modest increase in β-catenin signaling present in AXIN1-mutant cells may impose a selective advantage, for example by more readily inducing proliferation, survival or maintenance of stem cell characteristics. Nevertheless, it is clear that AXIN1 mutation by itself is not a strong driver of liver tumorigenesis. Deletion of *AXIN1* in the mouse liver only leads to few tumors with late onset^11,12^, implying that other oncogenic hits are required to successfully initiate tumor formation. This notion is also supported by the minimal changes we observe in the AXIN1-repaired SNU449 cells. Apparently, this cell line does not depend anymore on the AXIN1 mutation to grow efficiently, at least using the culture conditions that we tested thus far. RNA sequencing analysis also shows that besides β-catenin signaling no routes previously linked to AXIN1 are clearly affected, e.g. MYC, SMAD3 and TP53^39–42^. As reported by others, it is also clear that AXIN1-mutant HCCs follow a different route to tumorigenesis than the ones carrying oncogenic β-catenin mutations, and may more heavily depend on the activation of other signaling pathways^12^.

In our HCC panel, tankyrase inhibition led to some AXIN1 and/or AXIN2 accumulation in all lines tested, except Hep3B. In accordance with previous reports investigating β-catenin mutant CRC lines^24–26^, the β-catenin mutant HCC cell lines retain a high level of nuclear β-catenin signaling. Significant reductions in β-catenin signaling are observed in several of the AXIN1/non-mutant lines tested. This reduction is however not sufficient to significantly affect their growth in a short-term MTT-assay. This seems to contradict our previous study where all HCC cell lines were inhibited after siRNA-mediated β-catenin knockdown^14^. However, in this latter study we reached more than 80% reduction in total β-catenin protein levels, which is in strong contrast to basically unaltered levels in our current study.

Using a colony formation assay we observe significant reductions in colony numbers in 7 out of 9 lines, albeit modest in some (Hep3B, PLC/PRF/5, and SNU449). Not in all cases can this be easily linked to effects on β-catenin signaling, like the strong inhibitory effect on colony formation we observe for HepG2 and SNU398, despite retaining high levels of signaling. For other lines like Huh7, reduced β-catenin signaling clearly associates with reduced colony formation, while this may be somewhat the case for PLC/PRF/5 and HepaRG. Tankyrases have however also been linked to several other cellular processes such as telomere maintenance, Hippo signaling, mitosis and DNA strand break repair^30,33–35^, all processes that may affect colony formation when altered by tankyrase inhibition. Therefore, the reduced colony numbers that we observe for some lines, are likely explained by a combination of factors, in which the correct explanation will be different for each cell line or tumor under investigation. Another factor to take into account when evaluating the mechanism of tankyrase inhibition, are the off-target effects of various inhibitors, e.g. on other PARP enzymes^24,36^. These will become more apparent at higher concentrations, and ideally studies should be performed at inhibitor concentrations that just lead to a (sub)maximum accumulation of the tankyrase enzymes. Overall our study suggests that tankyrase inhibition at such concentrations is unlikely to contribute to HCC treatment as monotherapy exclusively through reduction of β-catenin signaling. A potential exception could be the rare subset of HCCs carrying APC mutations leading to high level signaling of wild-type β-catenin^9,48,49^.

In conclusion, using a panel of nine HCC cell lines we observe that all three AXIN1-mutant lines display a clearly increased activity in a β-catenin reporter assay. Repair of the AXIN1 mutation in SNU449 confirms this observation as it results in reduced activation of the β-catenin target gene *AXIN2*. In the three non-mutant lines reducing *AXIN1* levels also in all cases leads to enhanced signaling. Overall, these analyses show that AXIN1 mutation or inactivation leads to enhanced β-catenin signaling in HCC, questioning the strong statements that have been made in this regard. We also show that AXIN1 and AXIN2 work synergistically to control β-catenin signaling. While in the AXIN1-mutant lines, AXIN2 is solely responsible for keeping signaling in check, in the non-mutant lines both AXIN proteins contribute to β-catenin regulation to varying levels. Lastly, at concentrations that inhibit tankyrase activity some HCC lines were clearly affected in colony formation, but in most cases apparently independent from effects on β-catenin signaling.

## Methods

### Cell lines

CTNNB1-mutant HepG2, Huh6, SNU398, AXIN1-mutant Hep3B, PLC/PRF/5, SNU449 and non-mutant HepaRG, Huh7, SNU182 HCC and CRC cell line SW480 were cultured as reported previously^14^. The term “non-mutant” is used throughout the paper to indicate that these lines do not contain mutations in genes known to be linked to β-catenin signaling. Identity of all cell lines and clones thereof, was confirmed by the Erasmus Molecular Diagnostics Department, using Promega Powerplex-16 STR genotyping in October 2018. All cell lines tested negative for mycoplasma. Supplemental Table S1 provides official cell line names, RRID-numbers and gene mutations related to β-catenin signaling.

### Reagents

XAV939 and IWR-1 were purchased from Sigma-Aldrich. Antibodies used: β-catenin (610154, BD Transduction Laboratories), phospho-β-catenin (Ser33/37) (#2009, Cell Signaling Technology), AXIN1 (#2087 and #3323 Cell Signaling Technology; AF3287 R&D systems), AXIN2 (#2151, Cell Signaling Technology), Tankyrase-1/2 (sc-365897, Santa Cruz), β-actin (sc-47778, Santa Cruz). Anti-rabbit or anti-mouse IRDye-conjugated secondary antibodies (LI-COR Biosciences, Lincoln, USA) and anti-rabbit-HRP (P044801-2, Dako) were used for western blot analysis.

### β-catenin reporter assays

The β-catenin reporter assays were performed basically as previously described^46^. In short, we plated 5 × 10^4^ cells per well on 24-well plates, which were transfected with 250 ng Wnt Responsive Element (WRE) vector and 10 ng CMV-Renilla using FuGENE HD or Lipofectamin 2000 Transfection Reagent. We measured luciferase activities and normalized the data for the transfection efficiency by using the Dual Luciferase Reporter Assay system. Following normalization, WRE/CMV-Renilla ratios are obtained. For the data presented in Supplemental Figs. S2, S8B and S9 also a Mutant Responsive Element (MRE) control vector was transfected in independent wells to obtain WRE/MRE ratios.

### MTT assay

After incubation with XAV939 for 72 h, cells were analyzed by MTT assay as previously reported^14^. The mean and standard error were calculated for each condition.

### Western blotting

Cells were lysed and run for fluorescent western blotting analysis as previously reported^14^. Results were visualized with Odyssey 3.0 software. For AXIN1, AXIN2 and pS33/37-β-catenin western blot analysis we used Immobilon ECL Ultra HRP substrate (MerckMillipore). Membranes for ECL detection were blocked and incubated using Immobilon Block-FL reagent (MerckMillipore). Original western blot images are presented in Supplemental Fig. S13.

### Immunocytochemistry

Cells were seeded on Nunc Lab-TekII CC2 Chamber Slides (Thermo-Fisher Scientific). After 16 h treatment with 1 µM XAV939 or DMSO, cells were washed with PBS, fixed in PBS-buffered 4% paraformaldehyde for 10 min, permeabilized with PBS-0.2% Triton X-100 solution for 5 min and blocked with PBS containing 3% BSA for 30 min. Samples were incubated with primary antibodies at room temperature for 1 h, followed by PBS-Tween 20 0.05% washes and incubation with appropriate secondary antibodies for 1 h. Primary antibodies were diluted as follows: AXIN1 (AF3287, 1:100); AXIN2 (#2151, 1:200); Tankyrase-1/2 (sc-365897, 1:200). The following secondary reagents were used: Donkey anti-Goat-Alexa 647 (#A-21447), Donkey anti-Rabbit-Alexa 488 (#A-21206), Donkey anti-Mouse-Alexa 594 (#A-21203); all from Invitrogen, at 1:500 dilution. Slides were mounted with Vectashield mounting medium with DAPI (H-1200, Vector Laboratories). Images were generated using a Zeiss LSM510META confocal electroscope.

### Quantitative real-time polymerase chain reaction

RNA isolation and qRT-PCR analysis were performed as described previously^8,14,50^. In short, RNA was isolated with a Machery-NucleoSpin RNA II kit (Bioké, Leiden, The Netherlands) according to the manufacturer’s instructions, and quantified using a Nanodrop ND-1000 (Wilmington, DE, USA). CDNA was prepared from total RNA using a cDNA Synthesis Kit (TAKARA BIO INC). Quantitative PCR was performed using Sensimix SYBRGreen (Applied Biosystems) or TaqMan (AXIN1; Hs00394718_m1, AXIN2; Hs00610344_m1, GAPDH; Hs02786624-g1) Gene Expression Assays (Applied Biosystems). Analyses were performed using the StepOne Real-Time PCR System and the StepOnev2.0 software (Applied Biosystems, Darmstadt, Germany). All expression levels are depicted relative to the expression of *GAPDH*. Primer sequences are provided in Supplemental Table S3.

### Gene knockdown by small interfering RNA (siRNA)

SiRNA-mediated gene knockdown was performed as reported previously^14,50^. Smartpool ON-TARGETplus siRNAs targeting *AXIN1*, *AXIN2* or *APC* were purchased from Dharmacon (Supplemental Table S4). The ON-TARGETplus Non-targeting siRNA #2 was used as negative control. Cells were reverse-transfected in a 24-well plate using a total of 0.8 µl DharmaFECT formulation 4 (Thermo-Fisher Scientific) and 25 nM of each siRNA per well. Following 72 h incubation, the effect of knock-down was tested by qRT-PCR. Alternatively, for combined siRNA/β-catenin reporter assays, the siRNAs were co-transfected with WRE or MRE vectors and CMV-Renilla using lipofectamin 2000, and measured after 48 h.

### Colony formation assay

After trypsinization, 500–2500 cells for each cell line were seeded in 6-well plates and were cultured in complete DMEM medium containing 1 µM or 5 µM XAV939, 5 µM IWR-1 or DMSO as control. For each treatment and cell line 6 wells were used. Medium was changed every three days. Two weeks later, the cells were washed with PBS, fixed in 4% PBS-buffered paraformaldehyde for 10 min and stained with crystal violet solution and counted (Gelcount, Oxford Optronix Ltd.)^51^.

### CRISPR/Cas9 mediated repair of *AXIN1* mutation in SNU449 cells

CRISPR/Cas9 repair of the AXIN1 mutations was performed as reported previously^8^. A single guide RNA (sgRNA) encompassing the homozygous c.2134C>T *AXIN1* mutation present in SNU449 cells was designed and cloned into pSpCas9(BB)-2A-GFP (PX458), a gift from Feng Zhang (Addgene plasmid # 48138) using standard procedures. To repair this mutation a single-stranded oligodeoxynucleotide (ssODN) was ordered as Ultramer (Integrated DNA Technologies). Both the sgRNA and ssODN are described in Supplemental Table S5. Transfections were performed with the Amaxa Cell Line Nucleofector-Kit-V (Lonza) and Nucleofector-IIb device according to the manufacturer’s instructions. In brief, 5 × 10^6^ SNU449 cells were cotransfected with 2 µg PX458 and 2 µg ssODN. After nucleofection, complete DMEM medium with 7.5 µM RAD51-stimulatory compound-1 (RS-1, Sigma-Aldrich) was used for cell culture. After 48 h, GFP positive cells were sorted by FACS and seeded as single cells in 96-well plates. DNA from clones grown successfully from single cells was isolated using the QuickExtract DNA Extraction Solution (Epicentre). For each clone, a PCR product encompassing the mutation was subjected to sequencing.

### RNA extraction, Ilumina library preparation and sequencing

The RNA sequencing procedure was performed as previously described^52^.Total RNA was isolated with the Machery-NucleoSpin RNA II kit (Bioké, Leiden, The Netherlands) and quantified using a Nanodrop ND-1000 (Wilmington, DE, USA). RNA quality was checked using a RNA Pico chip on the Agilent Bioanalyzer. Library was constructed and sequenced with an Illumina HiSeqTM2000 (GATC Biotech, Konstanz, Germany). Briefly, the mRNA was enriched using oligo-dT magnetic beads, followed by fragmentation (about 200 bp). Then first strand cDNA was synthesized using random hexamer-primers and the second strand was further synthesized in a reaction buffer including dNTPs, RNase H and DNA polymerase I. Double stranded cDNA was purified with magnetic beads. Then, the 3′-end single nucleotide A (adenine) was added and adapters were ligated to the fragments which were enriched by PCR amplification.

### RNA-sequencing analysis

RNA-seq data of control and *AXIN1*-repaired SNU449 samples (n = 3 each) was analyzed using UCSC human genome build hg38 and GENCODE annotation release 26 (GRCh38). FASTQC (v0.11.5)^53^ was applied on the single-end FASTQ files for quality control, both before and after running trimmomatic (v0.36)^54^, which removed TrueSeq adapter sequences. STAR (v2.5.3a) was used as aligner, with 2-pass mapping for each sample separately^55^. Mapping quality plot was generated and checked based on sambamba Flagstat (v0.6.7) statistics^56^. Count files, with the number of reads for each gene were created with subread FeatureCounts (1.5.2)^57^. Settings of different tools can be seen in Supplemental Table S6. R (version 3.4.3) was used for further statistics calculation and data visualizations. Differential expression analysis were performed with condition ‘Repaired’ (n = 3) versus ‘Mutated’ (n = 3) using the DESeq2 package (v1.18.1)^58^ and the Wald-test. A significance cut-off of 0.05 on the adjusted P-value was utilized, using the Benjamini–Hochberg procedure. The RNA-sequencing data from this study have been submitted to the Gene Expression Omnibus (GEO)-database under the accession number GSE119001.

### Statistical analysis

All results are presented as mean ± SD. Statistical analyses were carried out using software GraphPad Prism version 8.4.1 (GraphPad Software Inc., San Diego, California, USA). Differences were considered significant at a *P* value less than 0.05 (*P < 0.05, **P < 0.01, ***P < 0.001).

## Supplementary Information

Supplementary Tables.

Supplementary Figures.

## Data Availability

All data generated or analyzed during this study are included in this published article and its supplementary information files. The RNA-sequencing data from this study have been submitted to the Gene Expression Omnibus (GEO)-database under the Accession Number GSE119001.

## References

[1] Forner A, Llovet JM, Bruix J (2012). Hepatocellular carcinoma. Lancet.

[2] Sherman M (2010). Hepatocellular carcinoma: Epidemiology, surveillance, and diagnosis. Semin. Liver Dis..

[3] Dahmani R, Just PA, Perret C (2011). The Wnt/beta-catenin pathway as a therapeutic target in human hepatocellular carcinoma. Clin. Res. Hepatol. Gastroenterol..

[4] Albuquerque C, Bakker ER, van Veelen W, Smits R (2011). Colorectal cancers choosing sides. Biochim. Biophys. Acta Rev. Cancer.

[5] Zucman-Rossi J, Villanueva A, Nault JC, Llovet JM (2015). Genetic landscape and biomarkers of hepatocellular carcinoma. Gastroenterology.

[6] Wang W, Pan Q, Fuhler GM, Smits R, Peppelenbosch MP (2017). Action and function of Wnt/beta-catenin signaling in the progression from chronic hepatitis C to hepatocellular carcinoma. J. Gastroenterol..

[7] Network CGAR (2017). Comprehensive and integrative genomic characterization of hepatocellular carcinoma. Cell.

[8] Liu P (2020). Oncogenic mutations in armadillo repeats 5 and 6 of beta-catenin reduce binding to APC, increasing signaling and transcription of target genes. Gastroenterology.

[9] Schulze K (2015). Exome sequencing of hepatocellular carcinomas identifies new mutational signatures and potential therapeutic targets. Nat. Genet..

[10] Zucman-Rossi J (2007). Differential effects of inactivated Axin1 and activated beta-catenin mutations in human hepatocellular carcinomas. Oncogene.

[11] Feng GJ (2012). Conditional disruption of Axin1 leads to development of liver tumors in mice. Gastroenterology.

[12] Abitbol S (2018). AXIN deficiency in human and mouse hepatocytes induces hepatocellular carcinoma in the absence of beta-catenin activation. J. Hepatol..

[13] Satoh S (2000). AXIN1 mutations in hepatocellular carcinomas, and growth suppression in cancer cells by virus-mediated transfer of AXIN1. Nat. Genet..

[14] Wang W (2016). Blocking Wnt secretion reduces growth of hepatocellular carcinoma cell lines mostly independent of beta-catenin signaling. Neoplasia.

[15] Qiao Y (2019). Axis inhibition protein 1 (Axin1) deletion-induced hepatocarcinogenesis requires intact beta-catenin but not notch cascade in mice. Hepatology.

[16] Gilgenkrantz H, Perret C (2019). Letter to the editor: Comment on Qiao et al. Hepatology.

[17] Chen X, Monga SP, Calvisi DF (2019). Reply. Hepatology.

[18] Zhang Y (2011). RNF146 is a poly(ADP-ribose)-directed E3 ligase that regulates axin degradation and Wnt signalling. Nat. Cell Biol..

[19] Callow MG (2011). Ubiquitin ligase RNF146 regulates tankyrase and Axin to promote Wnt signaling. PLoS ONE.

[20] Thorvaldsen TE, Pedersen NM, Wenzel EM, Stenmark H (2017). Differential roles of AXIN1 and AXIN2 in Tankyrase inhibitor-induced formation of degradasomes and beta-catenin degradation. PLoS ONE.

[21] Mariotti L, Pollock K, Guettler S (2017). Regulation of Wnt/beta-catenin signalling by tankyrase-dependent poly(ADP-ribosyl)ation and scaffolding. Br. J. Pharmacol..

[22] Bao R (2012). Inhibition of tankyrases induces Axin stabilization and blocks Wnt signalling in breast cancer cells. PLoS ONE.

[23] Busch AM (2013). Evidence for tankyrases as antineoplastic targets in lung cancer. BMC Cancer.

[24] Huang SM (2009). Tankyrase inhibition stabilizes axin and antagonizes Wnt signalling. Nature.

[25] Tanaka N (2017). APC mutations as a potential biomarker for sensitivity to tankyrase inhibitors in colorectal cancer. Mol. Cancer Ther..

[26] Lau T (2013). A novel tankyrase small-molecule inhibitor suppresses APC mutation-driven colorectal tumor growth. Can. Res..

[27] Waaler J (2012). A novel tankyrase inhibitor decreases canonical Wnt signaling in colon carcinoma cells and reduces tumor growth in conditional APC mutant mice. Can. Res..

[28] Schatoff EM (2019). Distinct colorectal cancer-associated APC mutations dictate response to tankyrase inhibition. Cancer Discov..

[29] Ma L (2015). Tankyrase inhibitors attenuate WNT/beta-catenin signaling and inhibit growth of hepatocellular carcinoma cells. Oncotarget.

[30] Jia J (2017). Tankyrase inhibitors suppress hepatocellular carcinoma cell growth via modulating the Hippo cascade. PLoS ONE.

[31] Thorvaldsen TE (2017). Targeting tankyrase to fight WNT-dependent tumours. Basic Clin. Pharmacol. Toxicol..

[32] Martino-Echarri E, Brocardo MG, Mills KM, Henderson BR (2016). Tankyrase inhibitors stimulate the ability of tankyrases to bind axin and drive assembly of beta-catenin degradation-competent axin puncta. PLoS ONE.

[33] Nagy Z (2016). Tankyrases promote homologous recombination and check point activation in response to DSBs. PLoS Genet..

[34] Chang P, Coughlin M, Mitchison TJ (2005). Tankyrase-1 polymerization of poly(ADP-ribose) is required for spindle structure and function. Nat. Cell Biol..

[35] Smith S, Giriat I, Schmitt A, de Lange T (1998). Tankyrase, a poly(ADP-ribose) polymerase at human telomeres. Science.

[36] Lum L, Chen C (2015). chemical disruption of wnt-dependent cell fate decision-making mechanisms in cancer and regenerative medicine. Curr. Med. Chem..

[37] Bienz M (2014). Signalosome assembly by domains undergoing dynamic head-to-tail polymerization. Trends Biochem. Sci..

[38] Gammons M, Bienz M (2018). Multiprotein complexes governing Wnt signal transduction. Curr. Opin. Cell Biol..

[39] Arnold HK (2009). The Axin1 scaffold protein promotes formation of a degradation complex for c-Myc. EMBO J..

[40] Rui Y (2004). Axin stimulates p53 functions by activation of HIPK2 kinase through multimeric complex formation. EMBO J..

[41] Li Q (2009). Axin determines cell fate by controlling the p53 activation threshold after DNA damage. Nat. Cell Biol..

[42] Guo X (2008). Axin and GSK3-control Smad3 protein stability and modulate TGF-signaling. Genes Dev..

[43] Rebouissou S (2016). Genotype-phenotype correlation of CTNNB1 mutations reveals different β-catenin activity associated with liver tumor progression. Hepatology.

[44] Fodde R, Tomlinson I (2010). Nuclear beta-catenin expression and Wnt signalling: In defence of the dogma. J. Pathol..

[45] Buchert M (2010). Genetic dissection of differential signaling threshold requirements for the Wnt/beta-catenin pathway in vivo. PLoS Genet..

[46] van Veelen W (2011). beta-catenin tyrosine 654 phosphorylation increases Wnt signalling and intestinal tumorigenesis. Gut.

[47] Bakker ER (2013). beta-Catenin signaling dosage dictates tissue-specific tumor predisposition in Apc-driven cancer. Oncogene.

[48] Guichard C (2012). Integrated analysis of somatic mutations and focal copy-number changes identifies key genes and pathways in hepatocellular carcinoma. Nat. Genet..

[49] Kan Z (2013). Whole-genome sequencing identifies recurrent mutations in hepatocellular carcinoma. Genome Res..

[50] Li S (2020). Commonly observed RNF43 mutations retain functionality in attenuating Wnt/beta-catenin signaling and unlikely confer Wnt-dependency onto colorectal cancers. Oncogene.

[51] Theil AF (2017). Trichothiodystrophy causative TFIIEbeta mutation affects transcription in highly differentiated tissue. Hum. Mol. Genet..

[52] Wang W (2019). Oncogenic STRAP Supports Hepatocellular Carcinoma Growth by Enhancing Wnt/beta-Catenin Signaling. Mol Cancer Res.

[53] Schmieder R, Edwards R (2011). Quality control and preprocessing of metagenomic datasets. Bioinformatics.

[54] Bolger AM, Lohse M, Usadel B (2014). Trimmomatic: A flexible trimmer for Illumina sequence data. Bioinformatics.

[55] Dobin A (2013). STAR: Ultrafast universal RNA-seq aligner. Bioinformatics.

[56] Tarasov A, Vilella AJ, Cuppen E, Nijman IJ, Prins P (2015). Sambamba: Fast processing of NGS alignment formats. Bioinformatics.

[57] Liao Y, Smyth GK, Shi W (2014). featureCounts: An efficient general purpose program for assigning sequence reads to genomic features. Bioinformatics.

[58] Love MI, Huber W, Anders S (2014). Moderated estimation of fold change and dispersion for RNA-seq data with DESeq2. Genome Biol.

