# Supplementary material for: Evaluation of AXIN1 and AXIN2 as targets of tankyrase inhibition in hepatocellular carcinoma cell lines
Source: Sci Rep. 2021 Apr 2;11:7470. doi: 10.1038/s41598-021-87091-4 (PMC8018973; doi:10.1038/s41598-021-87091-4)
Supplement: Supplementary file 1 — Supplementary Tables. [file 41598_2021_87091_MOESM1_ESM.docx]

**Supplemental Tables**

**Evaluation of AXIN1 and AXIN2 as targets of tankyrase inhibition in hepatocellular carcinoma cell lines**

Wenhui Wang^1,2†^, Pengyu Liu^1,3†^, Marla Lavrijsen^1^, Shan Li^1^, Ruyi Zhang^1^, Shanshan Li^1^, Wesley S. van de Geer^4,5^, Harmen J. G. van de Werken^4,5^, Maikel P. Peppelenbosch^1^, Ron Smits^1,^*

1. Department of Gastroenterology and Hepatology, Erasmus MC-University Medical Center, Rotterdam, The Netherlands.

2. Department of Pharmacology, China Pharmaceutical University, 211198, Nanjing, China.

3. Shenzhen Key Laboratory of Viral Oncology, The Clinical Innovation & Research Centre, Shenzhen Hospital, Southern Medical University, Shenzhen, Guangdong Province, China.

4. Department of Urology, Erasmus MC Cancer Institute, Erasmus MC-University Medical Center, Rotterdam, The Netherlands.

5. Cancer Computational Biology Center, Erasmus MC Cancer Institute, Erasmus MC-University Medical Center, Rotterdam, The Netherlands.

†These authors contributed equally to this paper

**Supplemental Table S1.** Gene mutations of Wnt/β-catenin signaling components in HCC cell lines and CRC line SW480. Table also indicates official cell line name and its Research Resource Identifier (RRID) as available from the ExPASy Cellosaurus database. All *CTNNB1* mutations lead to a gain of function, while *AXIN1* and *APC* mutations are loss of function mutations.

| Cell line | RRID | Gene | AA alteration | Zygosity |
| --- | --- | --- | --- | --- |
| Hep-G2 | CVCL_0027 | *CTNNB1* | p.W25_I140 del | Heterozygous |
| HuH-6 | CVCL_4381 | *CTNNB1* | p.G34V | Heterozygous |
| SNU-398 | CVCL_0077 | *CTNNB1* | p.S37C | Heterozygous |
| Hep 3B2.1-7 | CVCL_0326 | *AXIN1* | p.R146* | Homozygous |
| PLC/PRF/5 | CVCL_0485 | *AXIN1* | p.(R373_M418 del) | Homozygous |
| SNU-449 | CVCL_0454 | *AXIN1* | p.R712* | Homozygous |
| Huh-7 | CVCL_0336 |  |  |  |
| HepaRG | CVCL_9720 |  |  |  |
| SNU-182 | CVCL_0090 |  |  |  |
| SW480 (CRC) | CVCL_0546 | *APC* | p.Q1338* | Homozygous |

**Supplemental Table S2** Genes significantly changed in expression in AXIN1 repaired SNU449 cells.

| **Gene** | **log2FoldChange** | **p-adjusted** |
| --- | --- | --- |
| COL11A1 | -9,48 | 0,0341 |
| PCDH7 | -9,46 | 0,0033 |
| MPV17L | -4,83 | 0,0006 |
| MGAT5B | -2,76 | 0,0009 |
| AXIN2 | -2,62 | 0,0217 |

**Supplemental Table S3**. Primer sequences used for qRT-PCR

| Gene | Forward Sequence(5^’^~3^’^) | Reverse Sequence(5’~3’) |
| --- | --- | --- |
| *AXIN1* | AACGACAGCGAGCAGCAGAG | AGCTTGTGACACGGCCCTGG |
| *AXIN2* | TATCCAGTGATGCGCTGACG | TTACTGCCCACACGATAAGG |
| *TNKS1* | CCTGGCAGATCCTTCAGCAA | TTGTAGCCCGCTGCTAGATG |
| *TNKS2* | TGCCAGGAGTGGCAATGAAG | TTTCTGCCATCACTTGCGTG |
| *APC* | GCGCTTACTGTGAAACCTGT | GAACACACACAGCAGGACAG |
| *GAPDH* | TGTCCCCACCCCCAATGTATC | CTCCGATGCCTGCTTCACTACCTT |

**Supplemental Table S4**. Individual target sequences for each ON-TARGETplus SMARTpool:

NM_181050, AXIN1 (Catalog ID: L-009625-00-0005)

J-009625-06 CGAGAGCCAUCUACCGAAA

J-009625-07 GGAAAGGUGUUGGCAUUAA

J-009625-08 GAGCAAGUUUCACCGAAGA

J-009625-09 GCAUCGUUGUGGCGUACUA

NM_004655, AXIN2 (Catalog ID: L-008809-00-0005)

J-008809-05 AGACGGUGCUCCCGAUGUA

J-008809-06 GCGCUAUGUUGGUGACUUG

J-008809-07 GAAAUGCGUGGAUACCUUA

J-008809-08 GCGAUCCUGUUAAUCCUUA

NM_000038, APC (Catalog ID: L-003869-00-0005)

J-003869-09 GAUGAUAUGUCGCGAACUU

J-003869-10 AUGAUAAGCUCCCAAAUAA

J-003869-11 GAGAAUACGUCCACACCUU

J-003869-12 GAACUAGAUACACCAAUAA

D-001810-10, ON-TARGETplus Non-targeting pool

UGGUUUACAUGUCGACUAA

UGGUUUACAUGUUGUGUGA

UGGUUUACAUGUUUUCUGA

UGGUUUACAUGUUUUCCUA

**Supplemental Table S5**. sgRNA and ssODN used for repair of *AXIN1* mutation in SNU449 cells.

The lowercase “g” will mutate the PAM sequence while retaining Leucine coding. Underlined and bold **G** will repair the *AXIN1* nonsense mutation.

| sgRNA | GGAGGAGGCGCGCTGACGTC |
| --- | --- |
|  |  |
| ssODN | GTAACCCCCAAGACCCACCCCACCCCACGACGCGGCCGTACCTCTGCTTGGAGGGTGCTCGGCTGGCTCTCTTTTCTTCCTCCTCgAGACGTC**G**GCGCGCCTCCTCCAGCTGGGTTAGGGGGTTGGG |

**Supplemental Table S6**. Tool settings for RNA sequencing analysis:

| **general** |  |
| --- | --- |
| ROOTFOLDER | ~/Documents/HCC_AXIN1/ |
| FASTQFOLDER | raw/fastq/ |
| STARINDEXFOLDER | ~/Documents/hg38_merge/ |
| GTFFILE | ~/Documents/GTF/gencode.v26.annotation.gtf |
| **trimmomatic** |  |
| call | trimmomatic -Xmx12G |
| scratch | 10000 |
| mem | 10000 |
| time | 235 |
| **single** |  |
| slidingwindow | 4 |
| threads | 2 |
| phred | -phred33 |
| mode | SE |
| minQual | 2 |
| rnaadapterfile | resources/TruSeq2-SE.fa |
| seedmismatches | 1 |
| palindrom | 30 |
| minlen | 50 |
| score | 7 |
| **samtools** |  |
| call | samtools |
| **flagstat** |  |
| scratch | 2000 |
| mem | 4000 |
| time | 20 |
| threads | 1 |
| **sambamba** |  |
| call | sambamba |
| **markdup** |  |
| scratch | 2000 |
| mem | 4000 |
| time | 20 |
| threads | 6 |
| **fastqc** |  |
| call | fastqc |
| scratch | 10000 |
| mem | 10000 |
| time | 60 |
| threads | 1 |
| **star** |  |
| call | STAR |
| readFilesCommand | zcat |
| outSAMtype | BAM SortedByCoordinate |
| outSAMstrandField | intronMotif |
| outFilterIntronMotifs | RemoveNoncanonicalUnannotated |
| chimSegmentMin | 12 |
| chimJunctionOverhangMin | 12 |
| alignSJDBoverhangMin | 10 |
| alignMatesGapMax | 200000 |
| alignIntronMax | 200000 |
| outFilterMultimapNmax | 20 |
| outFilterMismatchNMAX | 5 |
| outStd | BAM_SortedByCoordinate |
| outSamUnmapped | Within |
| alignEndsType | Local |
| chimOutType | WithinBAM |
| twopassMode | Basic |
| twopass1readsN | -1 |
| quantMode | GeneCounts |
| scratch | 15000 |
| mem | 15000 |
| time | 235 |
| threads | 20 |
| **featureCounts** |  |
| call | featureCounts |
| scratch | 1000 |
| mem | 1000 |
| time | 30 |
| threads | 12 |
| parametersSingle | -t exon -g gene_id --largestOverlap --primary --fracOverlap 0.8 |
