# Supplementary material for: Evaluation of AXIN1 and AXIN2 as targets of tankyrase inhibition in hepatocellular carcinoma cell lines
Source: Sci Rep. 2021 Apr 2;11:7470. doi: 10.1038/s41598-021-87091-4 (PMC8018973; doi:10.1038/s41598-021-87091-4)

Evaluation of AXIN1 and AXIN2 as targets of tankyrase inhibition in hepatocellular carcinoma cell lines

Wenhui Wang[1,2†], Pengyu Liu[1,3†], Marla Lavrijsen[1], Shan Li[1], Ruyi Zhang[1], Shanshan Li[1], Wesley S. van de Geer[4,5], Harmen J. G. van de Werken[4,5], Maikel P. Peppelenbosch[1], Ron Smits[1,*]

1. Department of Gastroenterology and Hepatology, Erasmus MC-University Medical Center, Rotterdam, The Netherlands.
2. Department of Pharmacology, China Pharmaceutical University, 211198, Nanjing, China.
3. Shenzhen Key Laboratory of Viral Oncology, The Clinical Innovation & Research Centre, Shenzhen Hospital, Southern Medical University, Shenzhen, Guangdong Province, China.
4. Department of Urology, Erasmus MC Cancer Institute, Erasmus MC-University Medical Center, Rotterdam, The Netherlands.
5. Cancer Computational Biology Center, Erasmus MC Cancer Institute, Erasmus MC-University Medical Center, Rotterdam, The Netherlands.

†These authors contributed equally to this paper

Supplemental Figure S1. QRT-PCR results for *APC*, *AXIN1* and *AXIN2* in siRNA experiments. *APC* and *AXIN1* RNA levels are efficiently knocked-down by their corresponding Dharmacon siRNA Smartpools. Interpretation of *AXIN2* knock-down efficiency is complicated by the fact that total *AXIN2* RNA levels are simultaneously downregulated by siRNA as well as upregulated by the emerging enhanced β-catenin signaling. This is especially the case for the AXIN1-mutant lines, as they solely depend on AXIN2 in the breakdown complex. We envisage the following will happen. Directly following transfection, the siRNA mediated reduction in *AXIN2* RNA levels present at that time in these cells, will result in reduced AXIN2 protein levels and a strongly increased β-catenin signaling. As a result both the reporter activity (Figure 2) as well as *AXIN2* RNA expression will be strongly increased. As the siRNA will not be 100% effective, this will temporarily lead to more AXIN2 protein contributing to enhanced β-catenin breakdown. Both reporter activity and *AXIN2* RNA production will diminish temporarily, after which this cycle will repeat itself. At the time of analysis, this may result in the apparent contradictory result that *AXIN2* RNA levels increase with *AXIN2* siRNA treatment. Expression levels are depicted relative to the housekeeping gene *GAPDH*.

Figure S1

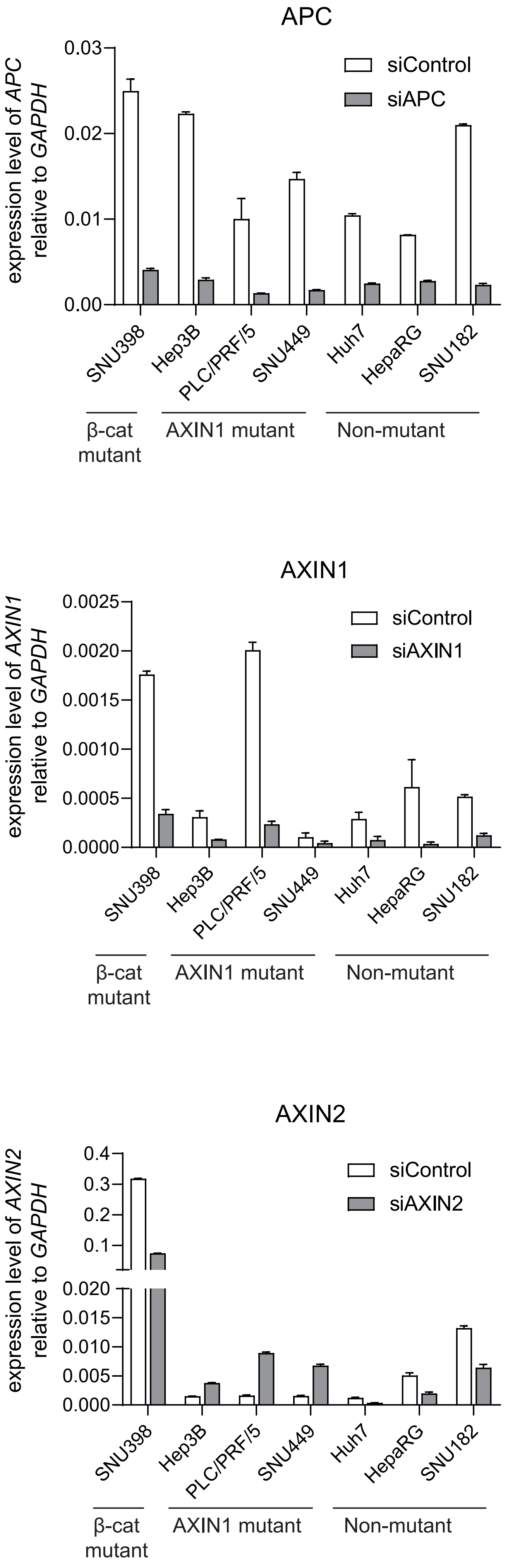
APC
expression level of APC relative to GAPDH
0.03
0.02
0.01
0.00
siControl
siAPC
SNU398
Hep3B
PLC/PRF/5
SNU449
Huh7
HepaRG
SNU182
β-cat mutant
AXIN1 mutant
Non-mutant
AXIN1
expression level of AXIN1 relative to GAPDH
0.0025
0.0020
0.0015
0.0010
0.0005
0.0000
siControl
siAXIN1
AXIN2
expression level of AXIN2 relative to GAPDH
0.4
0.3
0.2
0.1
0.020
0.015
0.010
0.005
0.000
siControl
siAXIN2

Supplemental Figure S2. Both AXIN1 and AXIN2 contribute to β-catenin signaling regulation in HCC cell lines. Independent experiments performed on relevant cell lines confirming the effects of *APC* or *AXIN1/2* knockdown in AXIN1- or non-mutant lines (A) Indicated cell lines were subjected to a β-catenin reporter assay after siRNA-mediated knockdown of *APC* or *AXIN2*. Both *APC* and *AXIN2* knockdown are equally effective in enhancing signaling in *AXIN1* mutant cells (Hep3B, PLC/PRF/5 and SNU449). *AXIN2* knockdown in the non-mutant lines (Huh7, HepaRG, SNU182) results in an incomplete increase in reporter activity when compared with *APC* knockdown. Absolute WRE/MRE β-catenin reporter ratios are shown following Renilla transfection normalization (mean ± SD, n=2, twice). Significance was tested using Mann-Whitney. (B) Indicated cell lines were subjected to a β-catenin reporter assay after siRNA-mediated knockdown of *AXIN1*, *AXIN2*, a combination thereof or *APC*. Combined *AXIN1/AXIN2* knockdown enhances reporter activity similar to *APC* knockdown. WRE/MRE ratios for the control siRNA were arbitrarily set to 1 for each cell line. All β-catenin reporter WRE/MRE ratios for the other siRNAs were normalized to this control (mean ± SD, n=2, twice). Only significant differences are shown using the Mann-Whitney test. Note the logarithmic Y-axis scales in both figures.

Figure S2

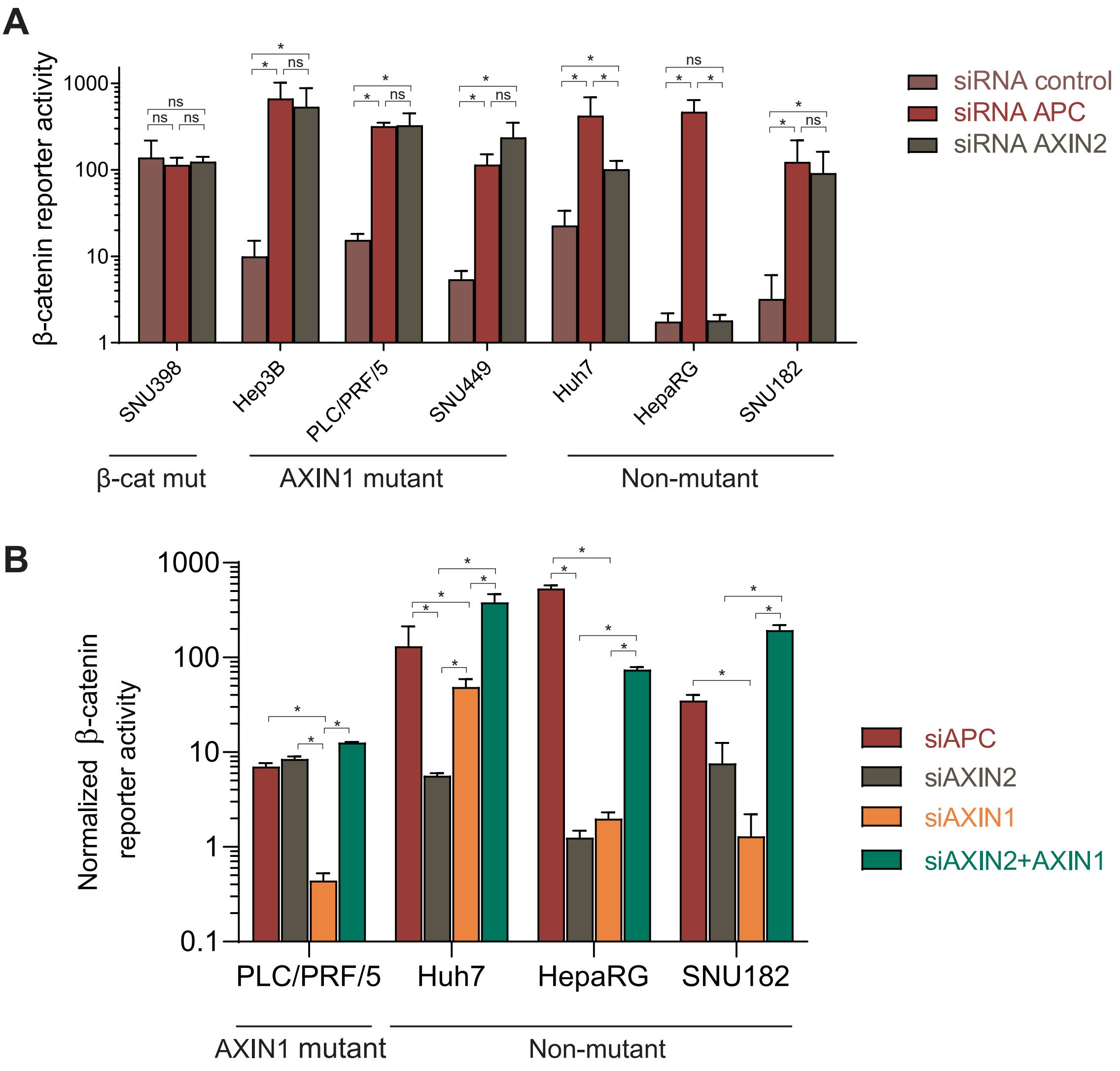
A
β-catenin reporter activity
1000
100
10
1
SNU398
Hep3B
PLC/PRF/5
SNU449
Huh7
HepaRG
SNU182
β-cat mut
AXIN1 mutant
Non-mutant
siRNA control
siRNA APC
siRNA AXIN2
B
Normalized β-catenin reporter activity
1000
100
10
1
0.1
PLC/PRF/5
Huh7
HepaRG
SNU182
AXIN1 mutant
Non-mutant
siAPC
siAXIN2
siAXIN1
siAXIN2+AXIN1

Supplemental Figure S3. *AXIN2* RNA levels following *APC*, *AXIN1*, or *AXIN2* knockdown. (A) *AXIN2* RNA expression is increased in all AXIN1- and non-mutant lines following *APC* knockdown. No change is observed in the β-catenin mutant SNU398 line. Depicted values are normalized to *AXIN2* levels observed for the control siRNA, which are arbitrarily set to 1. (B) *AXIN2* RNA expression is increased in all non-mutant lines following *AXIN1* knockdown, but not in the AXIN1- and β-catenin mutant lines. (C) *AXIN2* RNA expression following *AXIN2* knockdown. The interpretation problem described for the supplemental Figure S1 also applies here.

Figure S3 Observed fold changes in *AXIN2* RNA expression relative to siControl

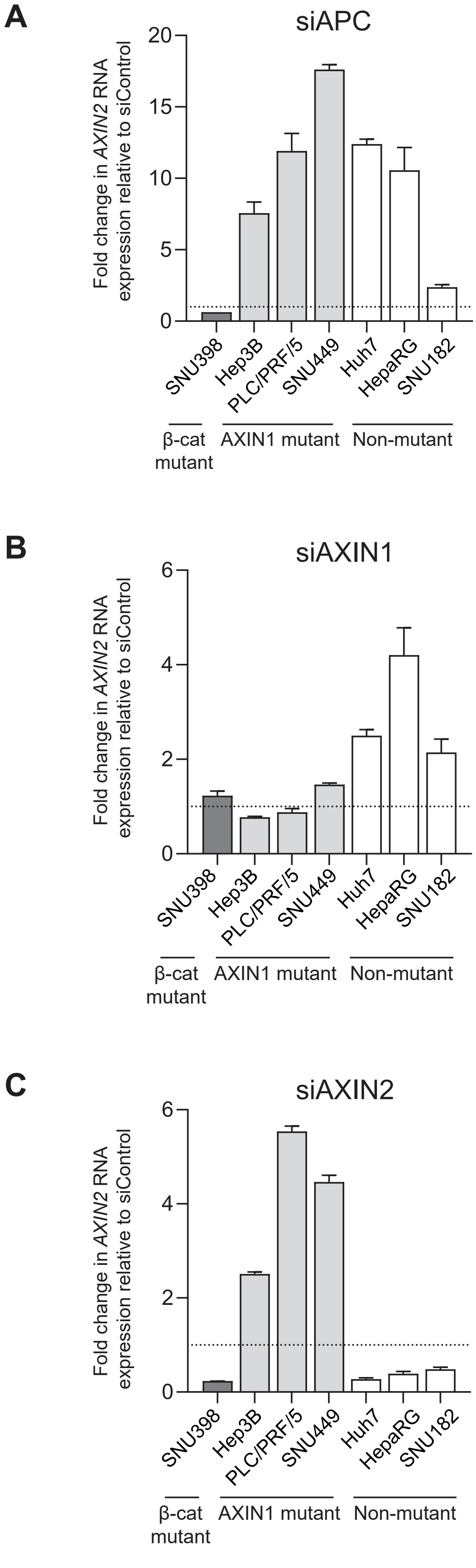

Supplemental Figure S4. Baseline levels of tankyrase-1/2 in HCC cell lines. (A) Western blotting assay showing the basal protein levels of tankyrase1/2. (B) QRT-PCR assay showing expression of *TNKS* and *TNKS2* in HCC cell lines (mean ± SD, n=2, twice). Expression levels are depicted relative to the housekeeping gene *GAPDH*.

Figure S4

A

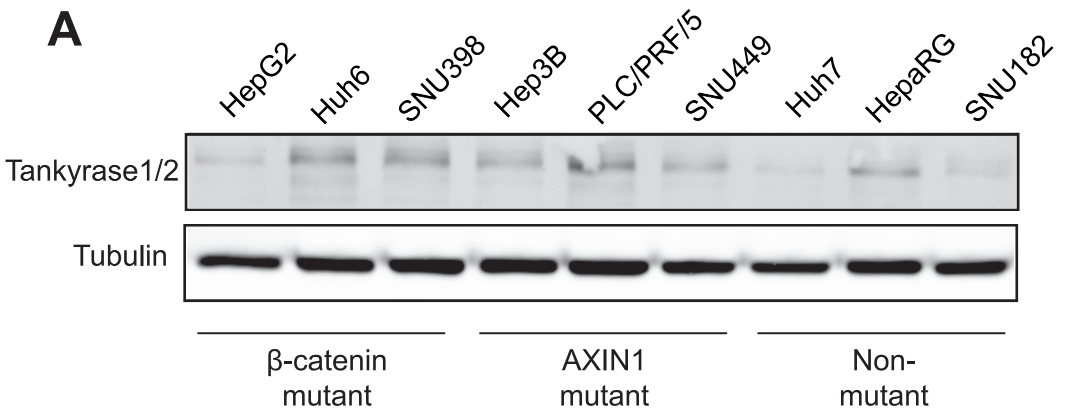
HepG2
Huh6
SNU398
Hep3B
PLC/PRF/5
SNU449
Huh7
HepaRG
SNU182
Tankyrase1/2
Tubulin
β-catenin mutant
AXIN1 mutant
Non-mutant

B

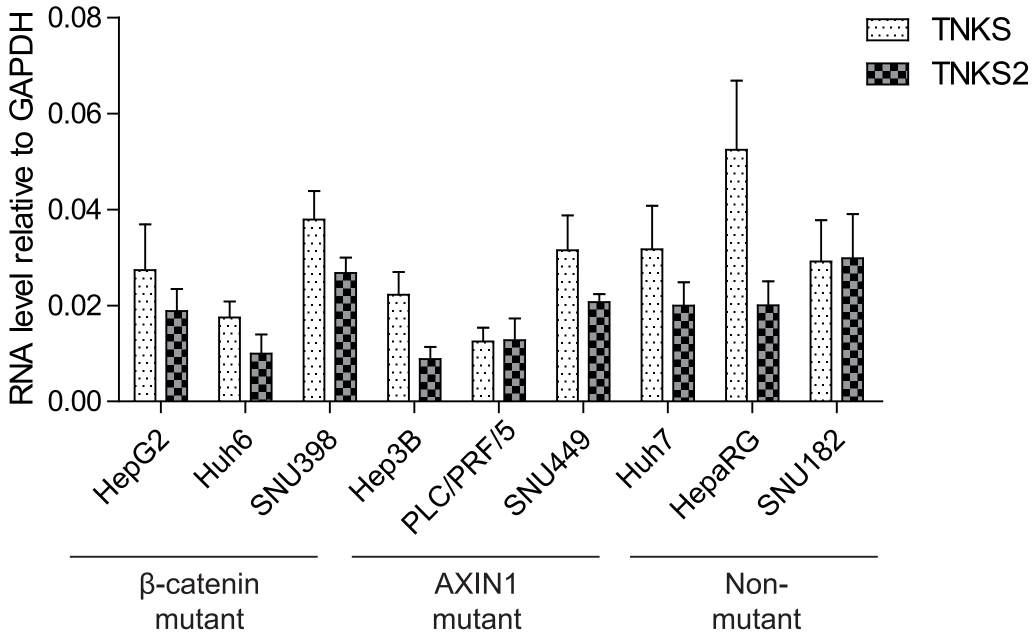
RNA level relative to GAPDH
0.08
0.06
0.04
0.02
0.00
TNKS
TNKS2
HepG2
Huh6
SNU398
Hep3B
PLC/PRF/5
SNU449
Huh7
HepaRG
SNU182
β-catenin mutant
AXIN1 mutant
Non-mutant

Supplemental Figure S5. Effect of tankyrase inhibitors validated by western blotting on SW480 cells. In SW480 cells XAV939 stabilizes tankyrase-1/2 and AXIN1/2 protein levels, increases pS33/37-β-catenin (p-β-catenin) while simultaneously reducing total β-catenin. SW480 cells were treated with indicated tankyrase inhibitors for 16 hours.

Figure S5

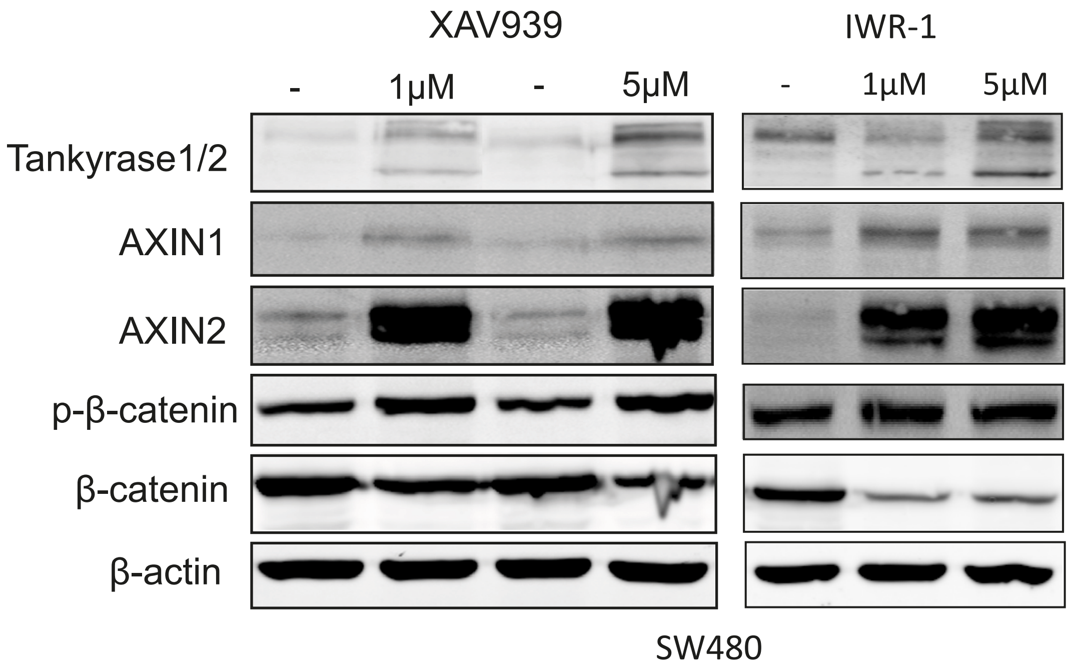
XAV939
IWR-1
-
1μM
-
5μM
-
1μM
5μM
Tankyrase1/2
AXIN1
AXIN2
p-β-catenin
β-catenin
β-actin
SW480

Supplemental Figure S6. Effect of tankyrase inhibition on indicated proteins. Independent experiment restricted to 1 and 5μM XAV-939 treatment for 24 hours. Western blotting assay showing levels of tankyrase-1/2, AXIN1/2, p33/37-β-catenin (pS33/37-β-cat) and total β-catenin. Again Tankyrase-1/2 efficiently accumulated in all cell lines, while variable responses are observed for AXIN1 and AXIN2. No clear change is observed in pS33/37-β-catenin and total β-catenin levels, except for Huh6 in which a modest change is seen.

Figure S6

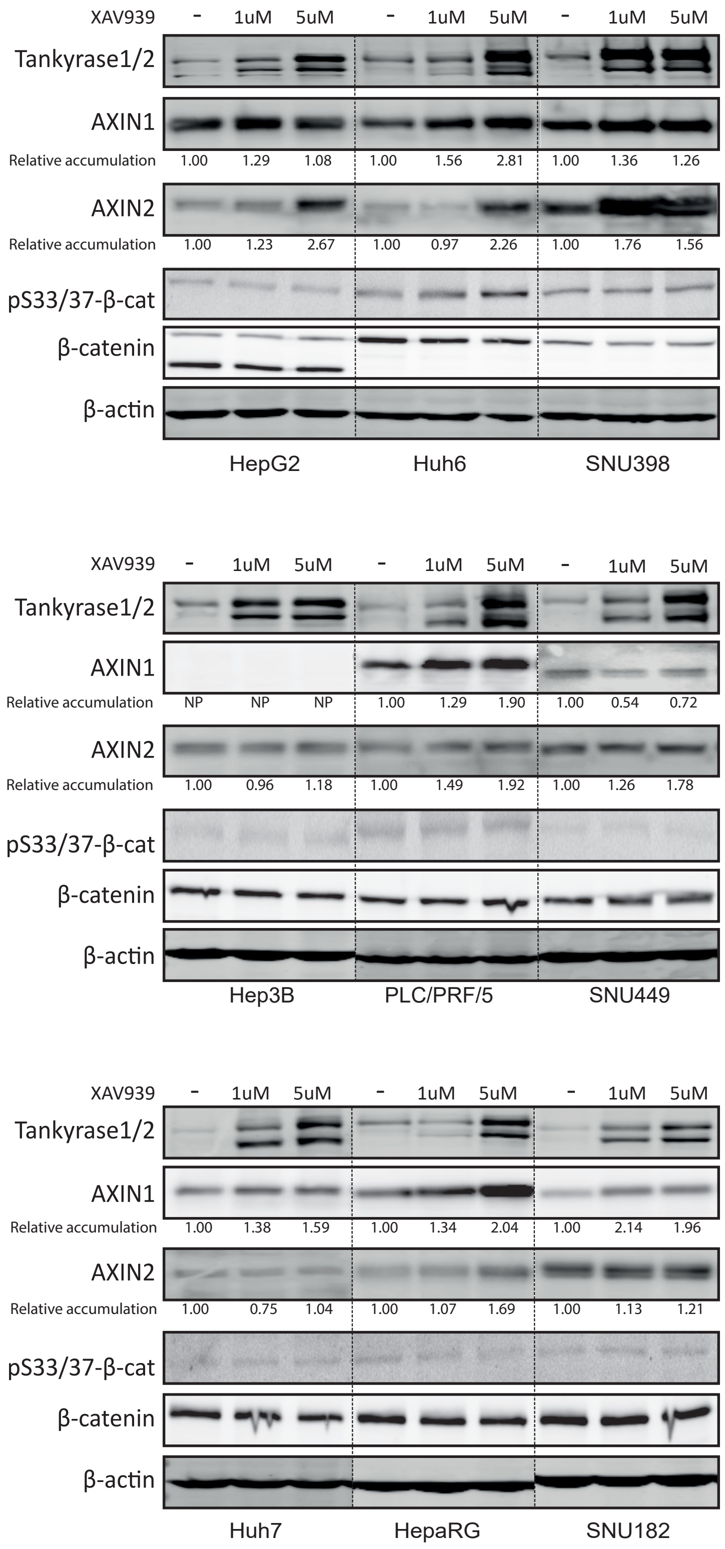
XAV939 - 1uM 5uM - 1uM 5uM - 1uM 5uM
Tankyrase1/2
AXIN1
Relative accumulation 1.00 1.29 1.08 1.00 1.56 2.81 1.00 1.36 1.26
AXIN2
Relative accumulation 1.00 1.23 2.67 1.00 0.97 2.26 1.00 1.76 1.56
pS33/37-β-cat
β-catenin
β-actin
HepG2
Huh6
SNU398
XAV939 - 1uM 5uM - 1uM 5uM - 1uM 5uM
Tankyrase1/2
AXIN1
Relative accumulation NP NP NP 1.00 1.29 1.90 1.00 0.54 0.72
AXIN2
Relative accumulation 1.00 0.96 1.18 1.00 1.49 1.92 1.00 1.26 1.78
pS33/37-β-cat
β-catenin
β-actin
Hep3B
PLC/PRF/5
SNU449
XAV939 - 1uM 5uM - 1uM 5uM - 1uM 5uM
Tankyrase1/2
AXIN1
Relative accumulation 1.00 1.38 1.59 1.00 1.34 2.04 1.00 2.14 1.96
AXIN2
Relative accumulation 1.00 0.75 1.04 1.00 1.07 1.69 1.00 1.13 1.21
pS33/37-β-cat
β-catenin
β-actin
Huh7
HepaRG
SNU182

Supplemental Figure S7. Immunofluorescence staining showing the abundance and subcellular localization of tankyrase-1/2 and both AXINs, following tankyrase inhibition. SW480 and Huh6 cells show a clear formation of Tankyrase1/2 and AXIN1/2-positive puncta. In PLC/PRF/5 cells Tankyrase1/2 accumulation is obvious, present for the mutant AXIN1 protein, but hardly discernible for AXIN2. Hep3B shows a weak formation of TNKS/2-positive puncta, while no AXIN1/2 can be observed. Original magnification is 63x. Cells were treated with 1µM XAV939 for 16h.

Figure S7

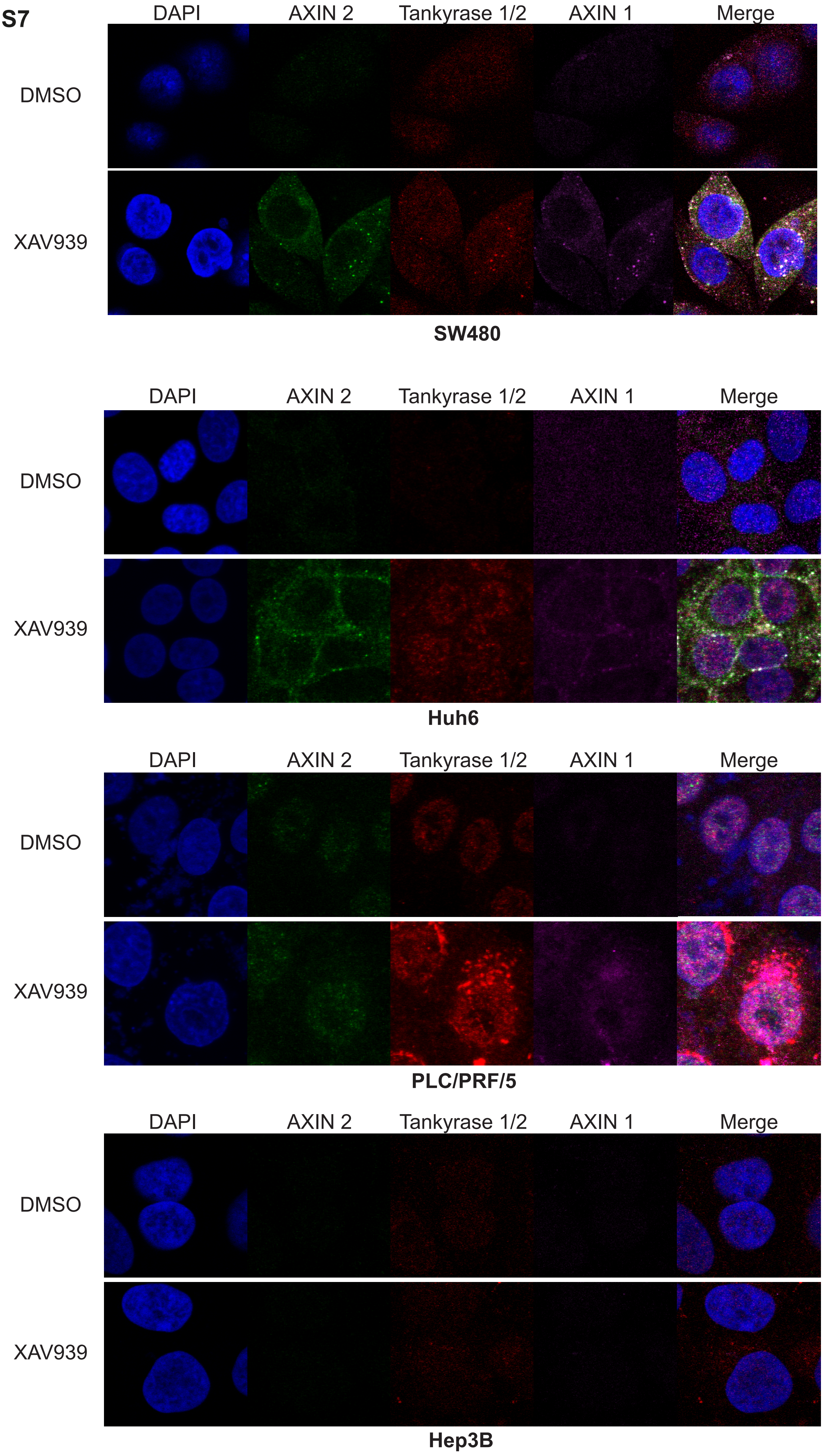
DAPI
AXIN 2
Tankyrase 1/2
AXIN 1
Merge
DMSO
XAV939
SW480
DAPI
AXIN 2
Tankyrase 1/2
AXIN 1
Merge
DMSO
XAV939
Huh6
DAPI
AXIN 2
Tankyrase 1/2
AXIN 1
Merge
DMSO
XAV939
PLC/PRF/5
DAPI
AXIN 2
Tankyrase 1/2
AXIN 1
Merge
DMSO
XAV939
Hep3B

Supplemental Figure S8. Absolute *AXIN2* RNA levels and β-catenin reporter values following tankyrase inhibition. Both these figures are shown to illustrate that despite the 2-3 fold reduction in *AXIN2* and/or β-catenin reporter levels observed in some of the lines, the absolute level of nuclear β-catenin signaling remains high in especially the CTNNB1- and AXIN1-mutant lines. (A) Absolute *AXIN2* RNA levels relative to *GAPDH,* following tankyrase inhibition. Note the logarithmic scale. Data and significance (not shown) are same as used for Figure 4 (mean ± SD, n=3, twice). (B) Independent β-catenin reporter assays performed using 1μM XAV939. For each cell line the absolute WRE/MRE ratios are depicted following CMV-Renilla transfection normalization. Showing the absolute values highlights the strongly increased β-catenin signaling present in β-catenin-mutant lines and intermediate levels in AXIN1-mutant lines, and shows that nuclear β-catenin signaling remains clearly present. Significant reductions using the Mann-Whitney test are observed in PLC/PRF/5 and Huh7 (mean ± SD, n=2, twice, *P<0.05; 48 hours treatment).

Figure S8

A

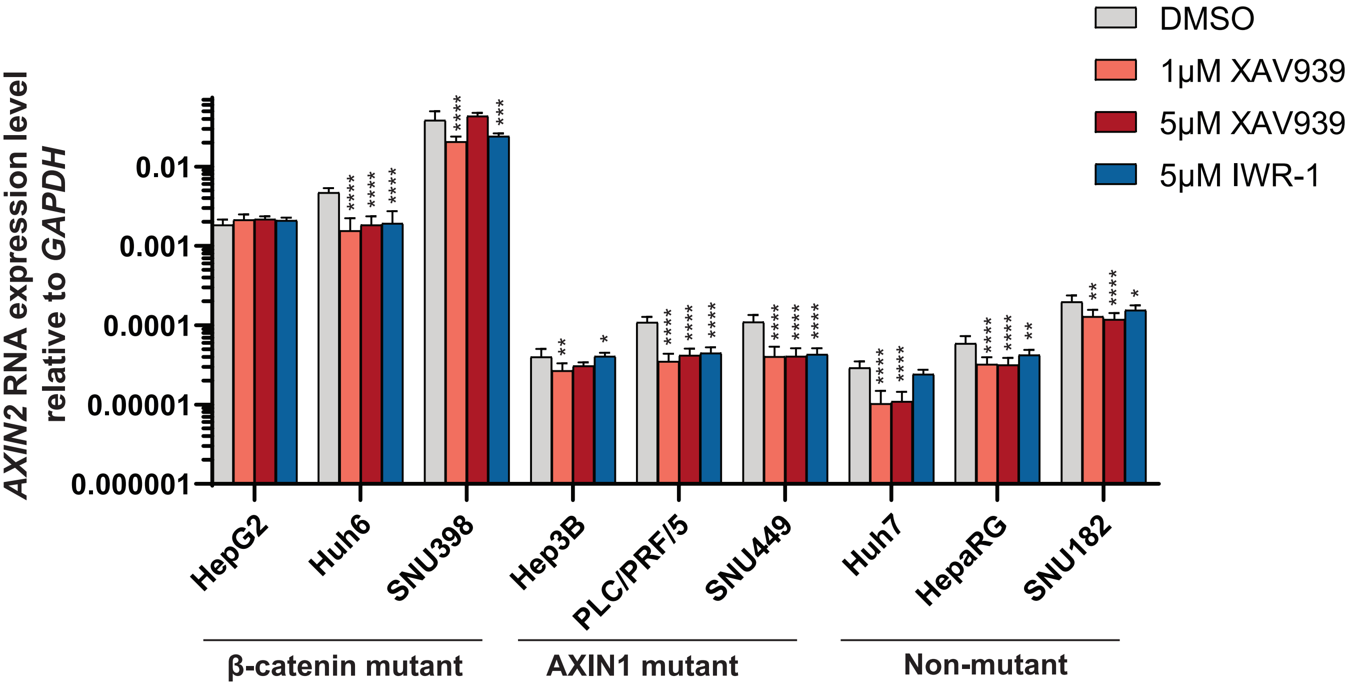
DMSO
1µM XAV939
5µM XAV939
5µM IWR-1
AXIN2 RNA expression level relative to GAPDH
0.01
0.001
0.0001
0.00001
0.000001
HepG2
Huh6
SNU398
Hep3B
PLC/PRF/5
SNU449
Huh7
HepaRG
SNU182
β-catenin mutant
AXIN1 mutant
Non-mutant

B

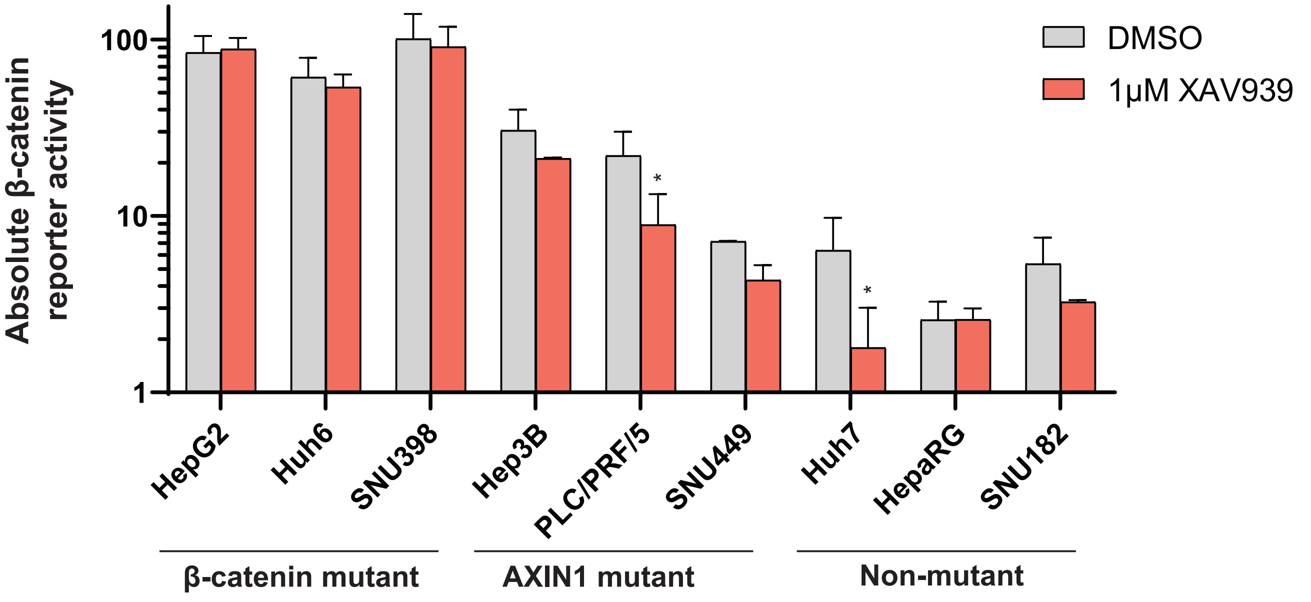
DMSO
1µM XAV939
Absolute β-catenin reporter activity
100
10
1
HepG2
Huh6
SNU398
Hep3B
PLC/PRF/5
SNU449
Huh7
HepaRG
SNU182
β-catenin mutant
AXIN1 mutant
Non-mutant

Supplemental Figure S9. Effect of 1μM XAV939 tankyrase inhibitor on β-catenin reporter activity. Data from Supplemental Figure S8 are shown here as fold changes relative to the DMSO control-treated samples.

Figure S9

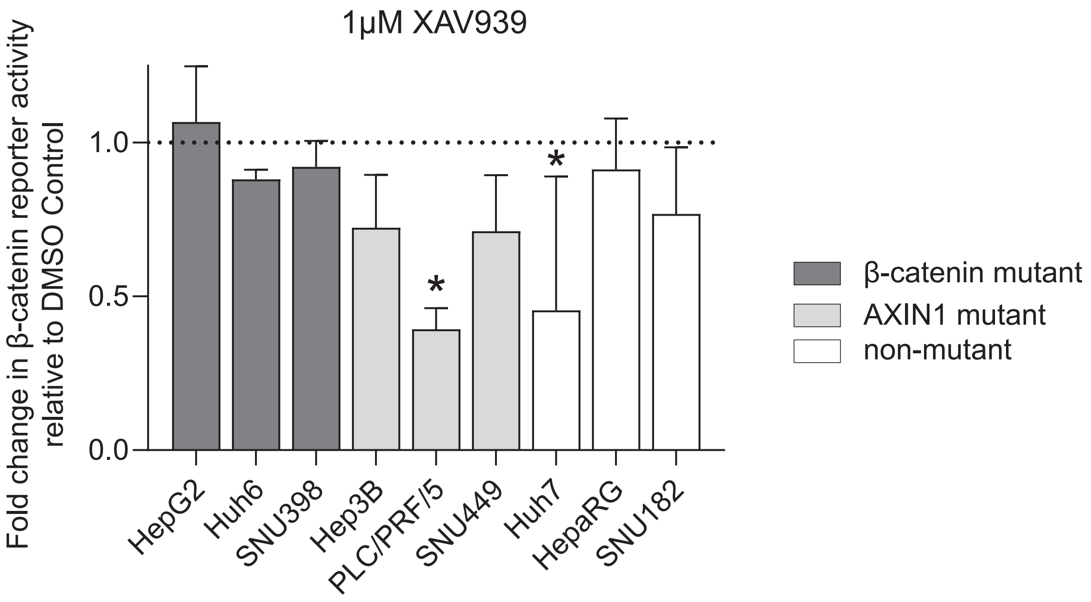

1µM XAV939
Fold change in β-catenin reporter activity relative to DMSO Control
1.0
0.5
0.0
*
*
HepG2
Huh6
SNU398
Hep3B
PLC/PRF/5
SNU449
Huh7
HepaRG
SNU182
β-catenin mutant
AXIN1 mutant
non-mutant

Supplemental Figure S10. Original images of colony formation assays. Colony formation for indicated cell lines was performed in 6-fold with DMSO, 1-5µM XAV939 or 5µM IWR-1 for approximately two weeks, after which the colonies were stained and counted (Gelcount, Oxford Optronix Ltd.). For all cell lines we plated 1000 cells per well except for SNU449 (500 cells), and HepG2, SNU398 and HepaRG (2500 cells). Medium was changed every three days.

Figure S10-1

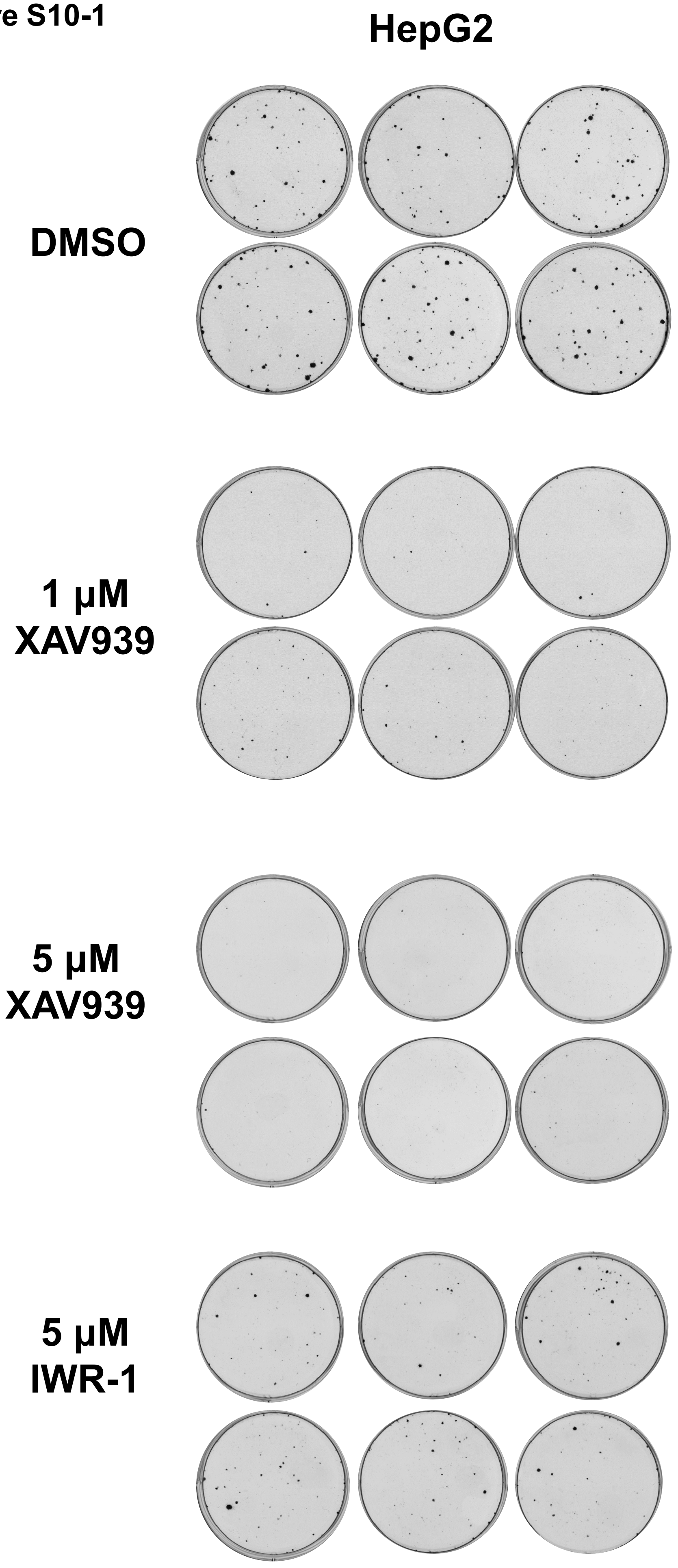

Figure S10-2

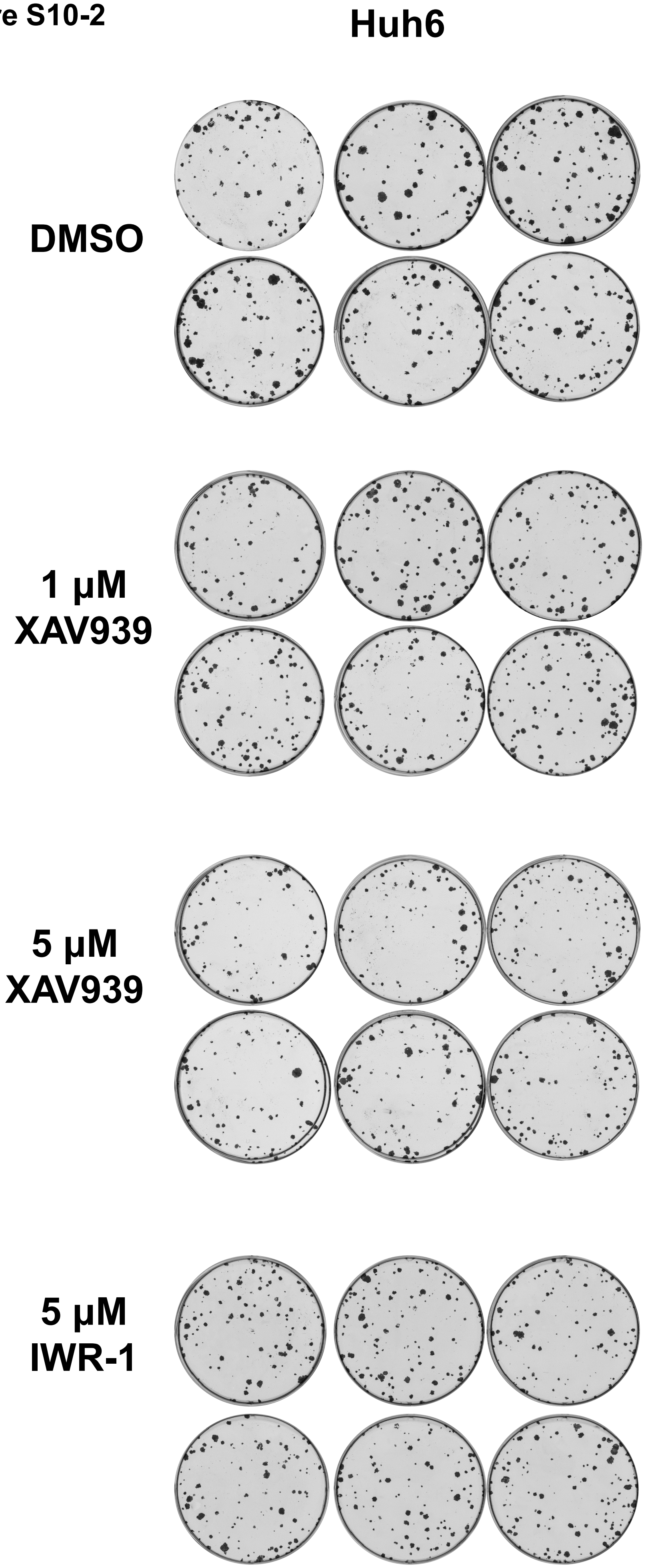

Figure S10-3

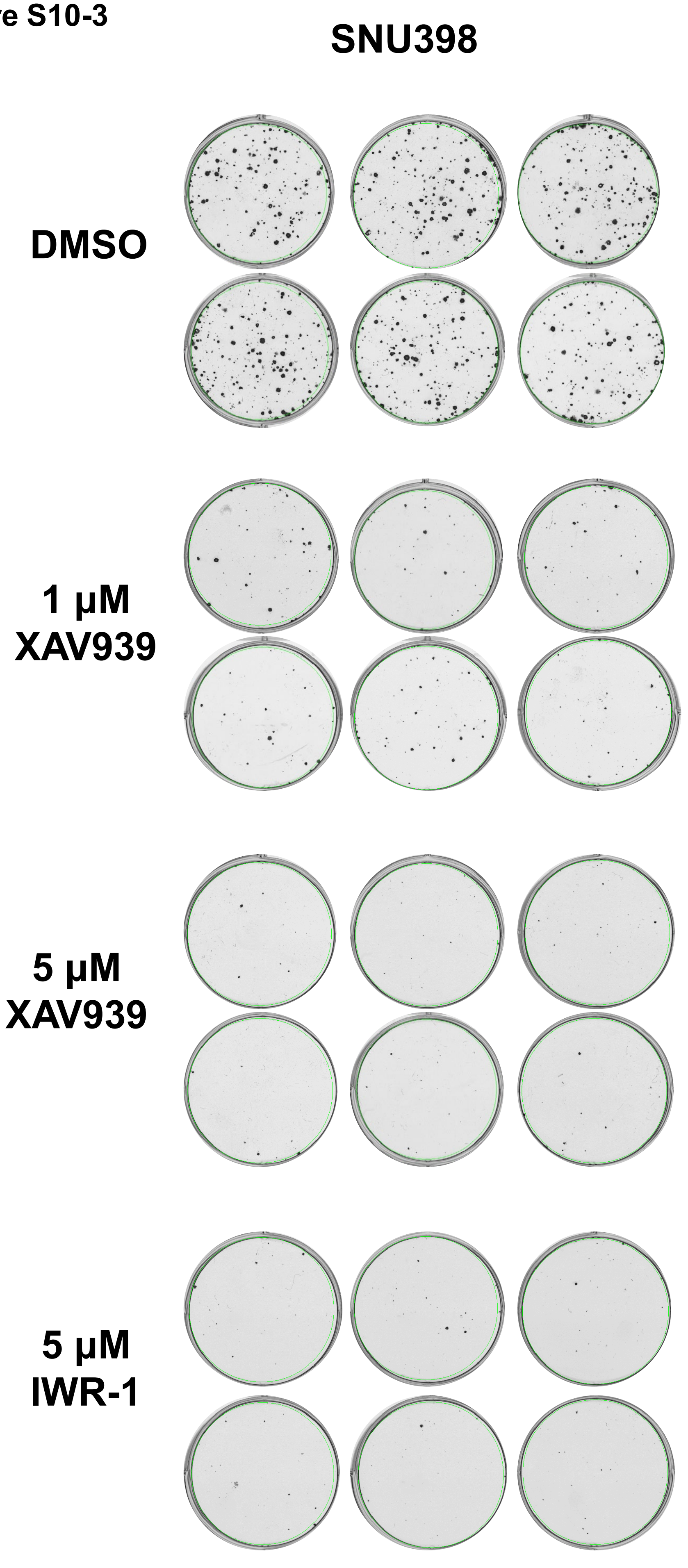

Figure S10-4

Hep3B

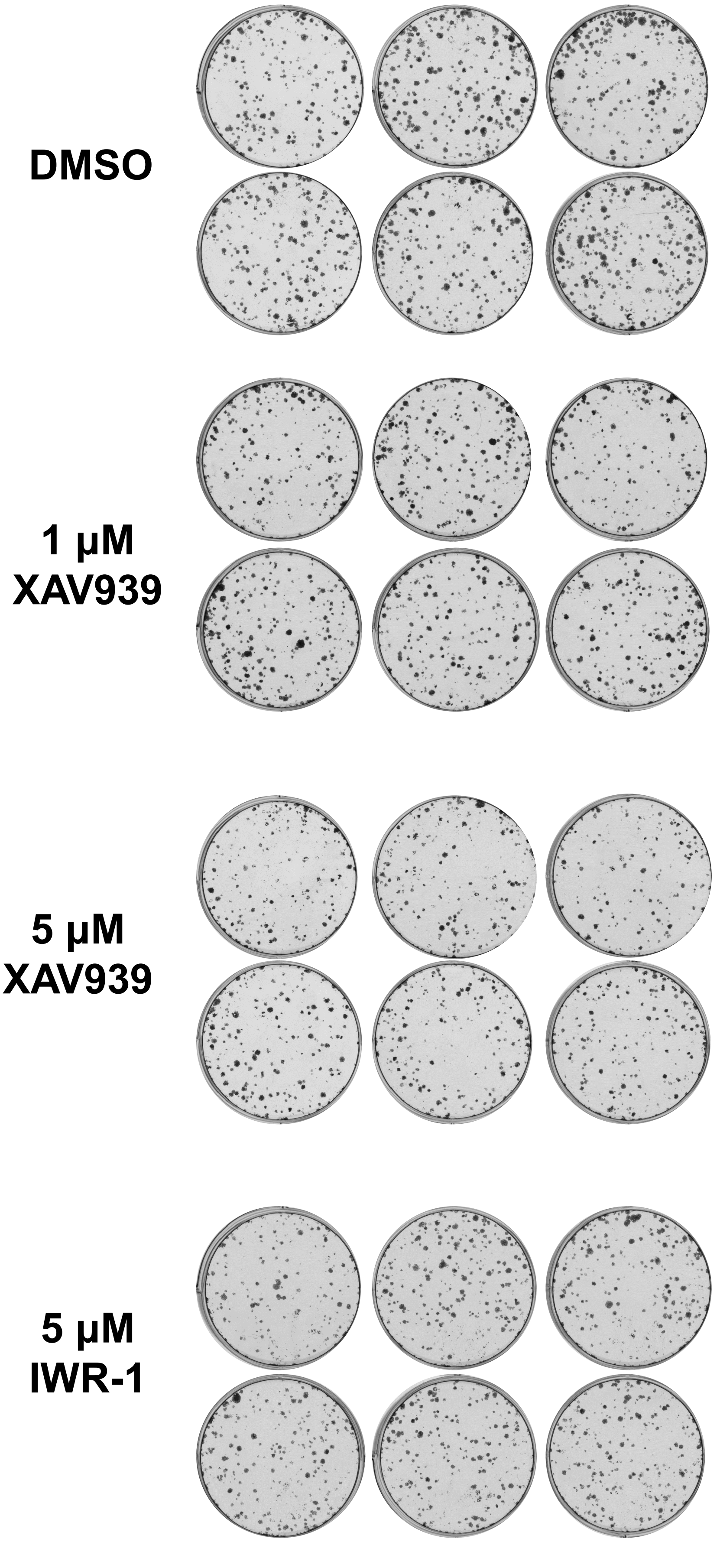

Figure S10-5

PLC/PRF/5

DMSO

1 µM XAV939

5 µM XAV939

5 µM IWR-1

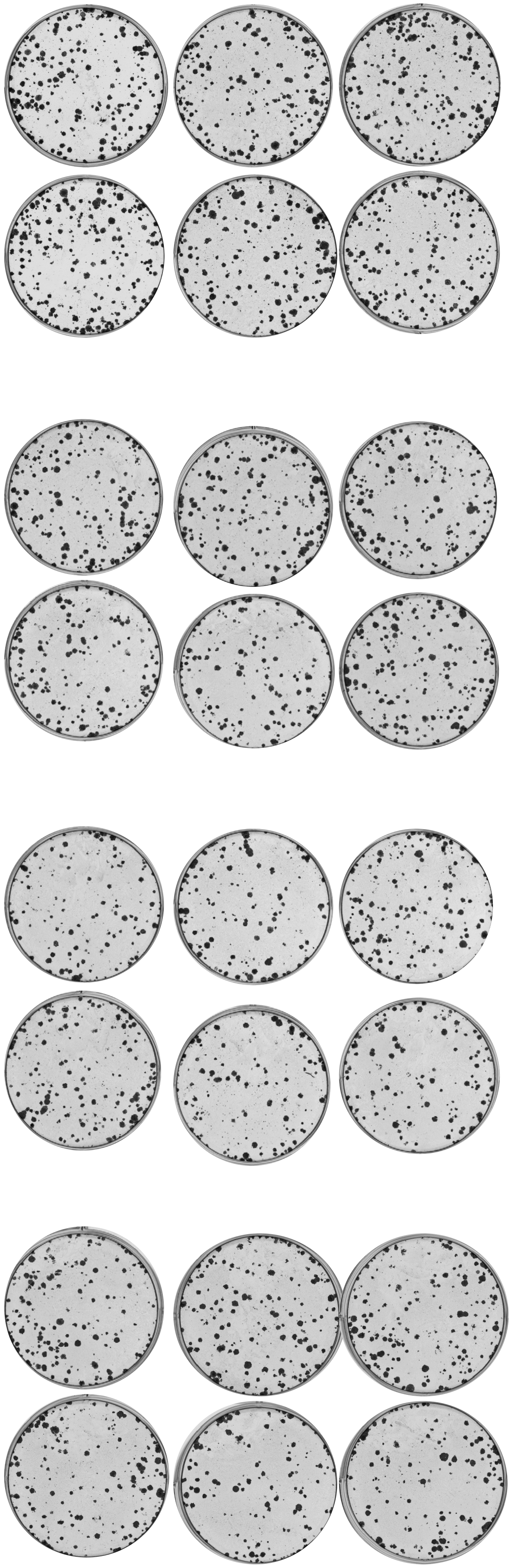

Figure S10-6

SNU449

DMSO

1 µM XAV939

5 µM XAV939

5 µM IWR-1

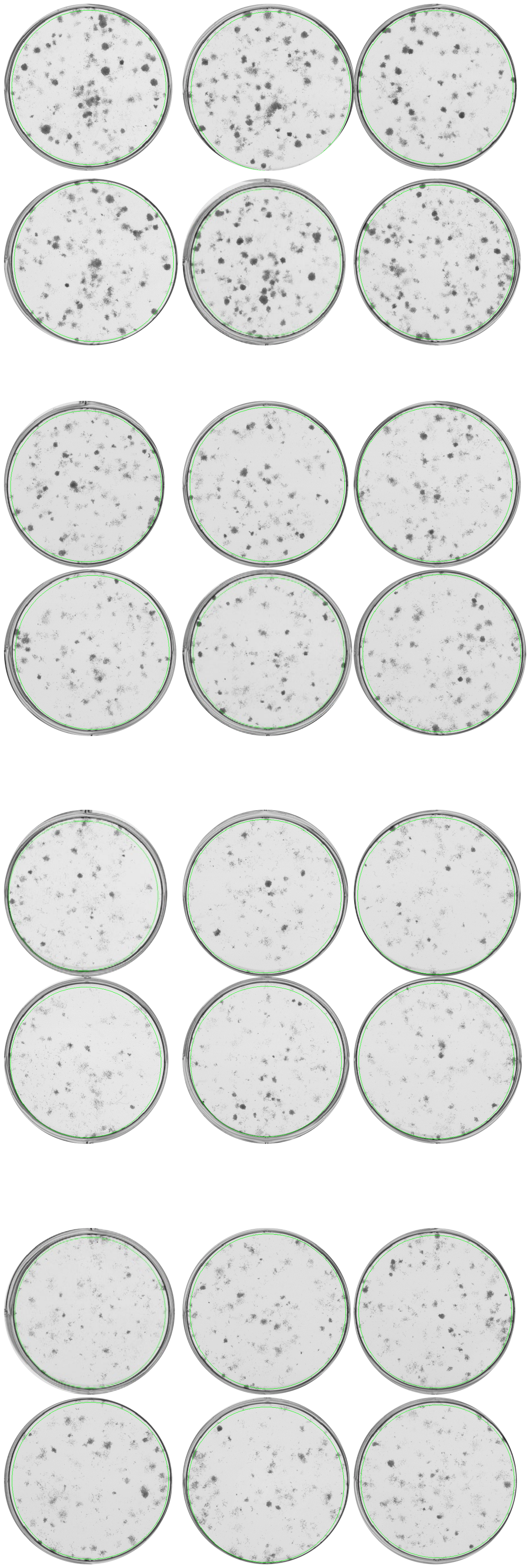

Figure S10-7

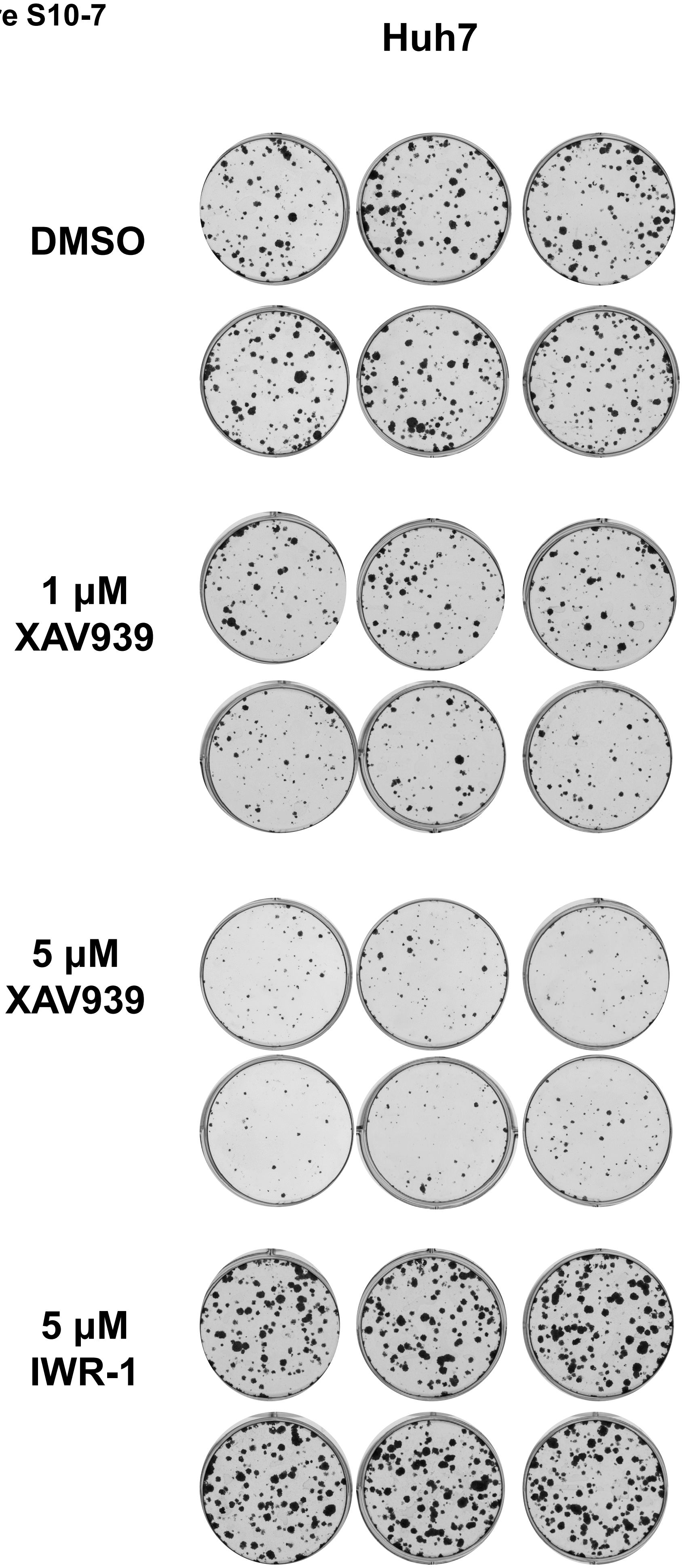

Figure S10-8

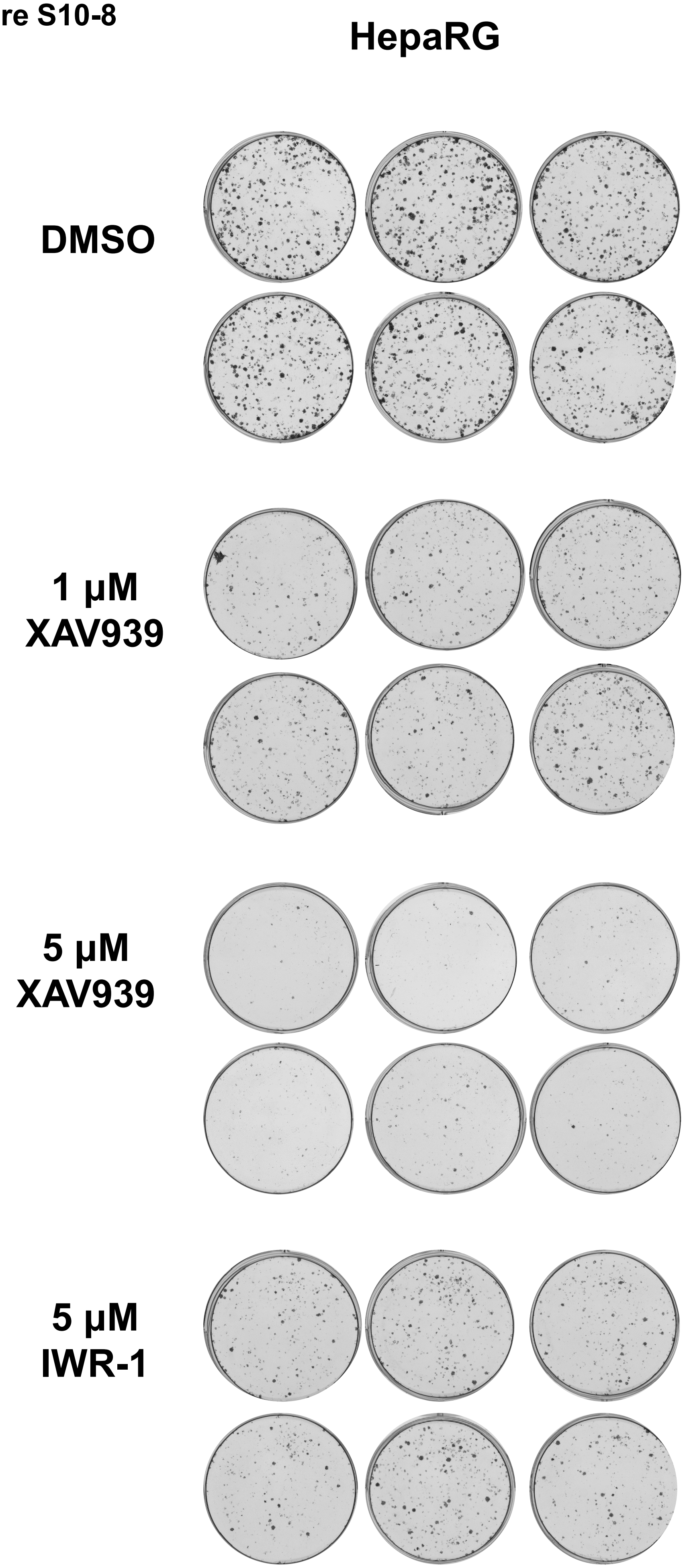

Figure S10-9

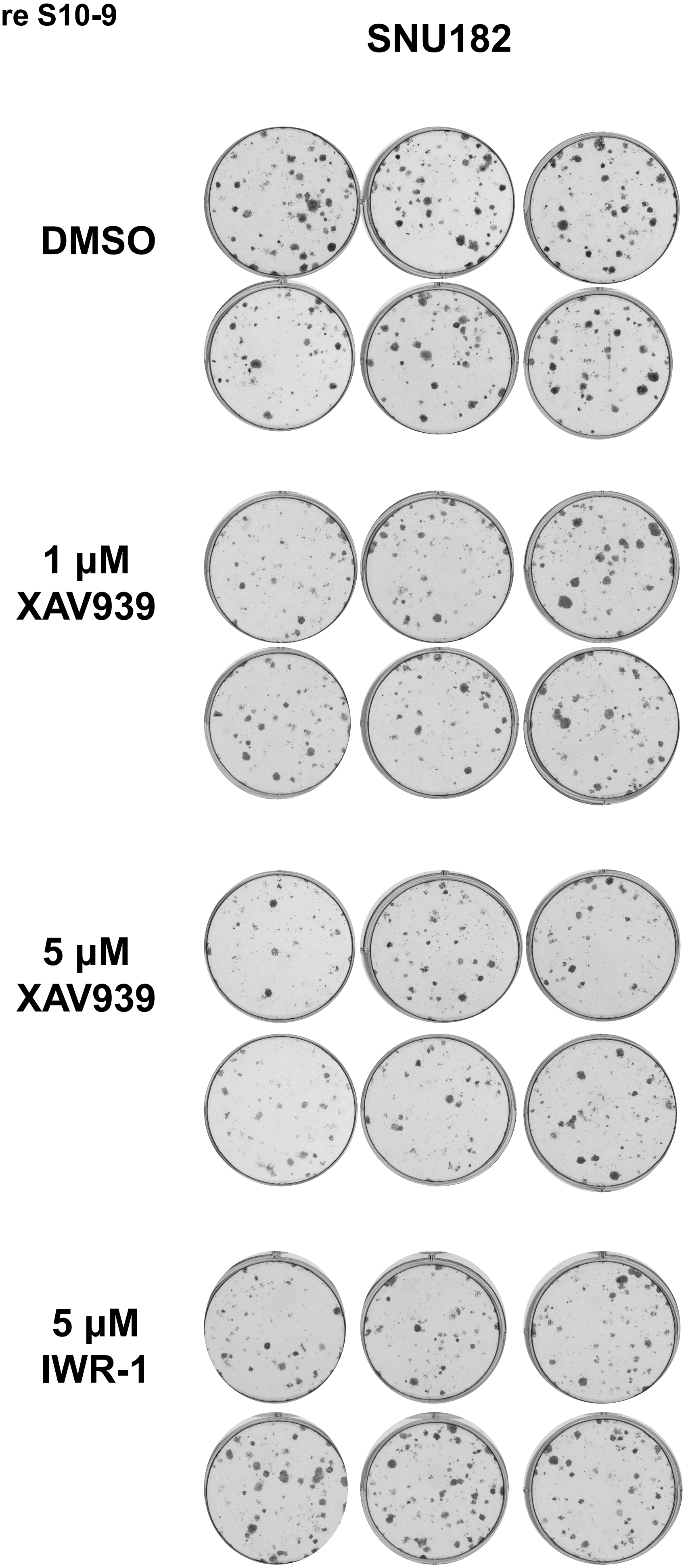

Supplemental Figure S11. Successful repair of the *AXIN1* mutation in SNU449 cells. (A) Western blot analysis shows the successful restoration of full-length (FL) AXIN1 protein expression in several independent clones (14-65). In control clones only the truncated protein is weakly visible. (B) QRT-PCR of *AXIN1* shows a 2-3 fold increased expression in all repaired clones. (C) *AXIN2* RNA expression is consistently down-regulated in all repaired clones. AXIN1/2 qRT-PCRs were normalized to the value obtained for clone 2, which was arbitrarily set to 1.

Figure S11

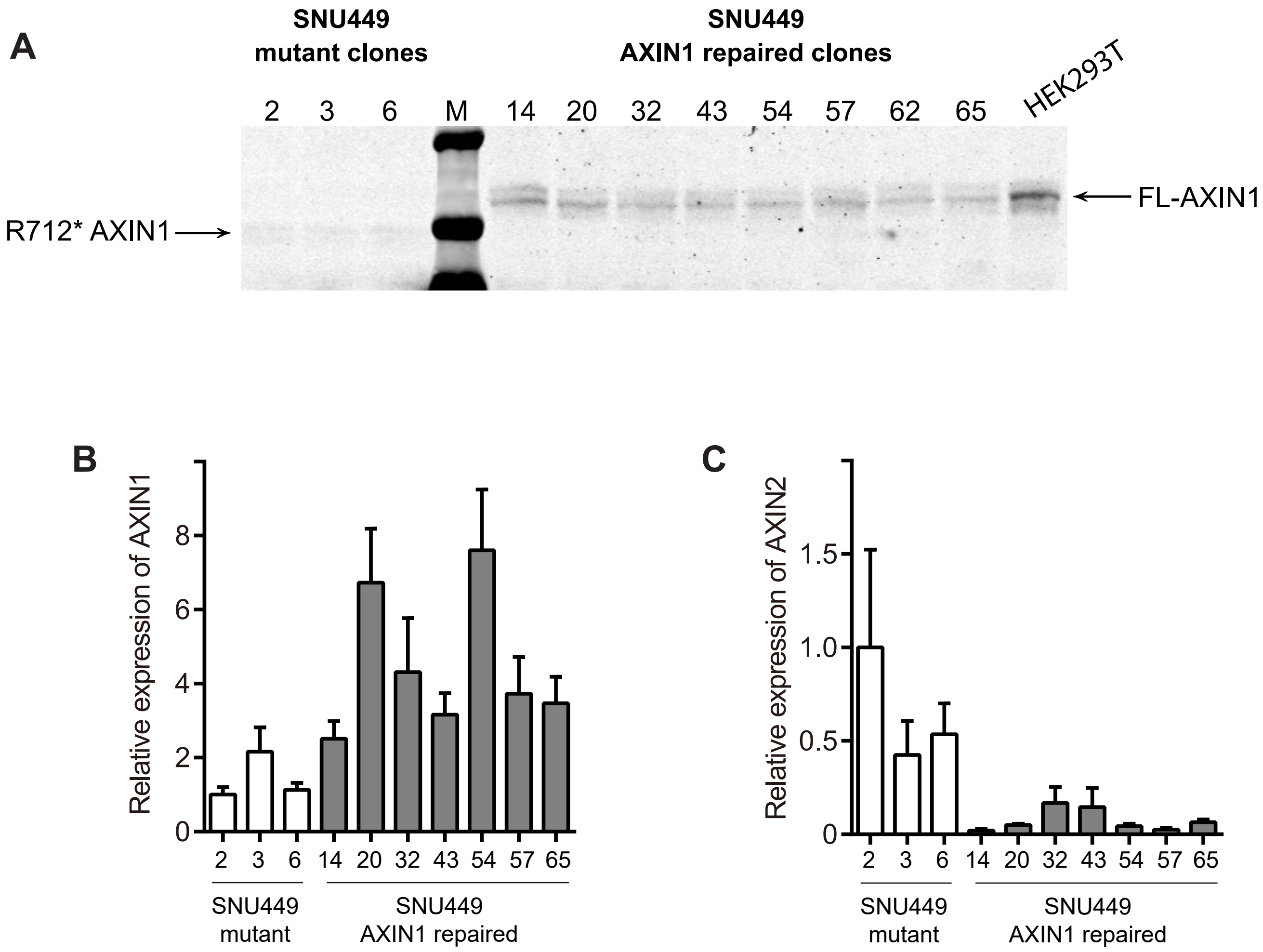
A
SNU449
mutant clones
SNU449
AXIN1 repaired clones
2 3 6 M 14 20 32 43 54 57 62 65 HEK293T
R712* AXIN1
FL-AXIN1
B
Relative expression of AXIN1
0 2 4 6 8
2 3 6 14 20 32 43 54 57 65
SNU449
mutant
SNU449
AXIN1 repaired
C
Relative expression of AXIN2
0 0.5 1.0 1.5
2 3 6 14 20 32 43 54 57 65
SNU449
mutant
SNU449
AXIN1 repaired

Supplemental Figure S12. Comparison of cell doubling times of AXIN1-repaired SNU449 clones. For each clone 2500 cells were seeded in 96-wells in 6-fold, followed by MTT measurements at days 2, 3, 4, 5 and 6. The three AXIN1-repaired clones show comparable doubling times with the parental SNU449 cell line (marked with red circle). Among the clones retaining the original AXIN1 mutation, more variation is observed. This variation was consistently observed in three independent experiments, i.e. clone 6 in all cases showed the shortest doubling times, while clone 3 was in all cases the slowest growing clone. Overall, this assay shows that the AXIN1-repaired clones retain normal cell doubling times.

Figure S12

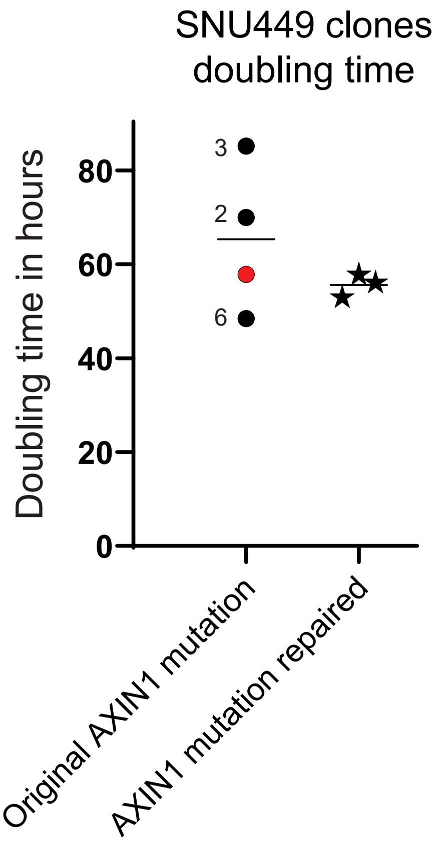
SNU449 clones
doubling time
Doubling time in hours
80
60
40
20
0
3
2
6
Original AXIN1 mutation
AXIN1 mutation repaired

Supplemental Figure S13. Original western blot scans used for this publication.

Supplemental Figure S13

Original IB images for Figure 1A

HepG2-Huh6-SNU398-Hep3B-PLC/PRF/5-SNU449-Huh7-HepaRG-SNU182

C-Terminal Axin1 #2087 (ECL)

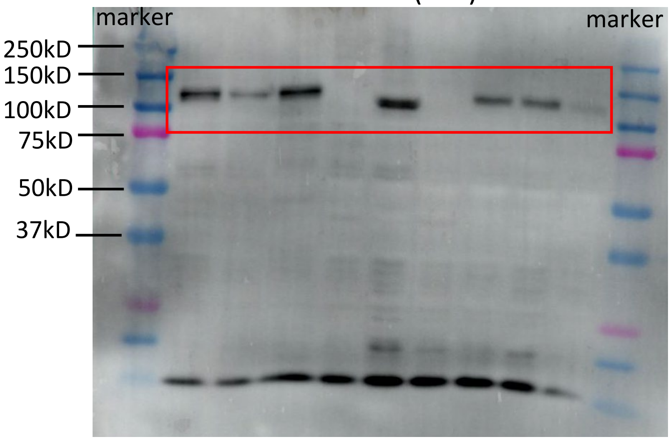

N-Terminal Axin1 #3323 (ECL)

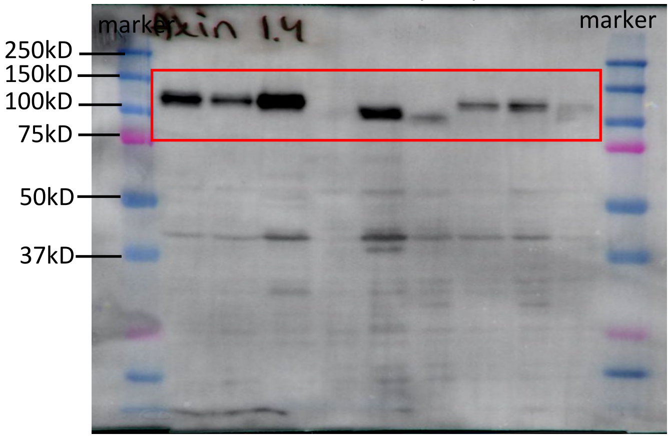

Axin2 (ECL)

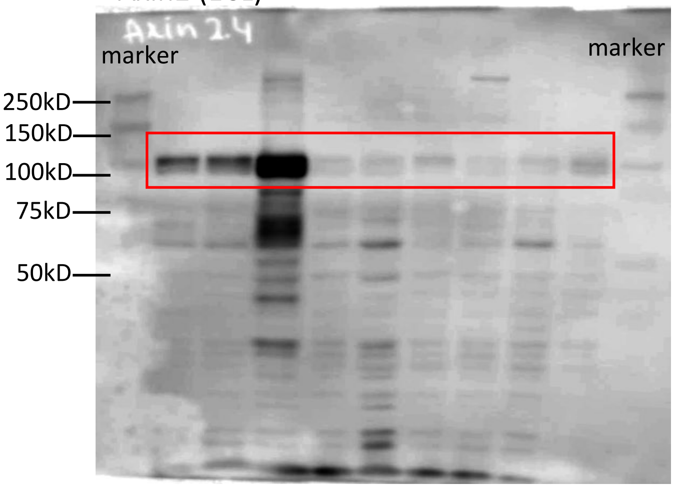

β-Actin (Fluorescence)

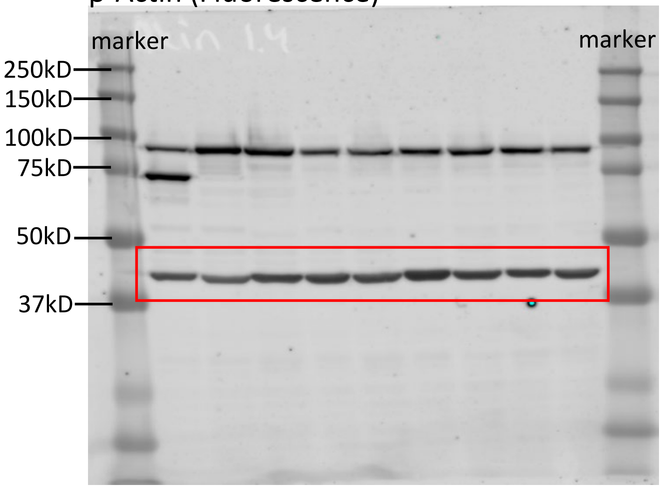

Original IB images for Figure 3: HepG2 – Huh6 – SNU398

XAV939 compound concentration : 0μM – 1μM – 5μM, IWR-1: 5μM.

N-Terminal Axin1 #3323 (ECL)

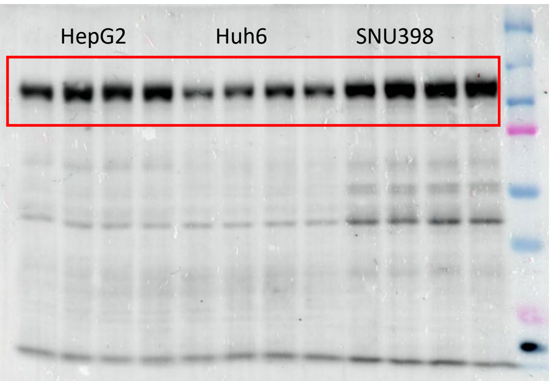

Axin2 (ECL)

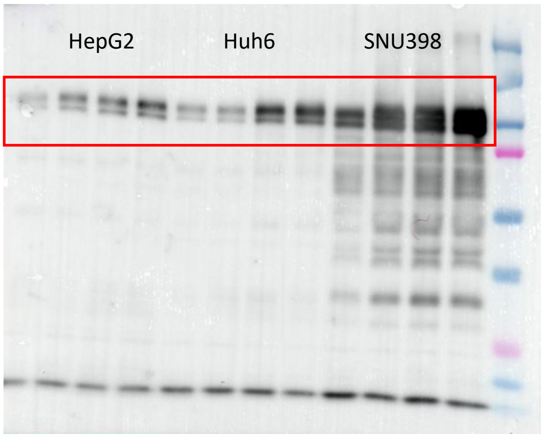

β -catenin (Fluorescence)

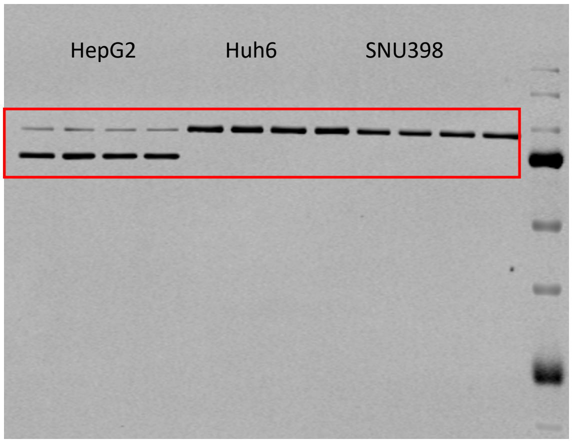

pS33/37 bcatenin (ECL)

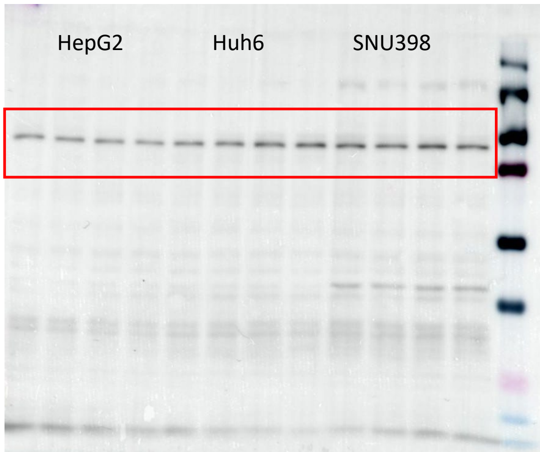

TNKS1/2 (up) and β -actin (Fluorescence)

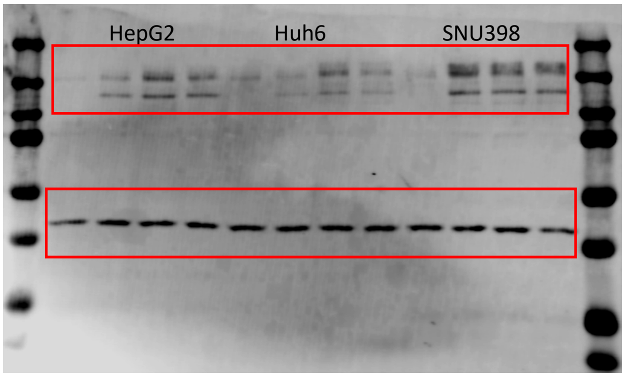

Original IB images for Figure 3: Hep3B – PLC – SNU449

XAV939 compound concentration : 0µM – 1µM – 5µM, IWR-1: 5µM.

N-Terminal Axin1 #3323 (ECL)

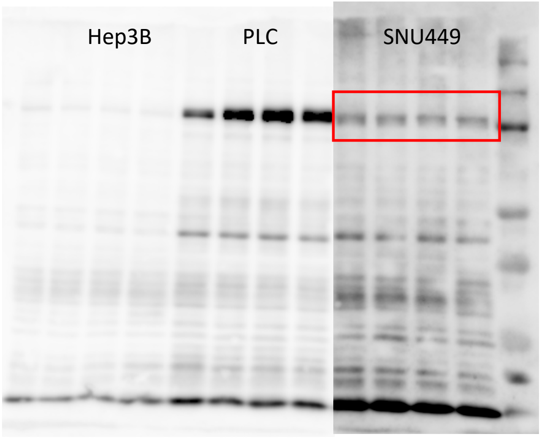

Axin2 (ECL)

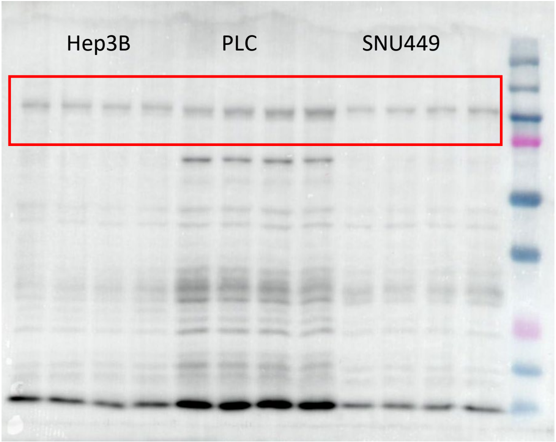

β -catenin (Fluorescence)

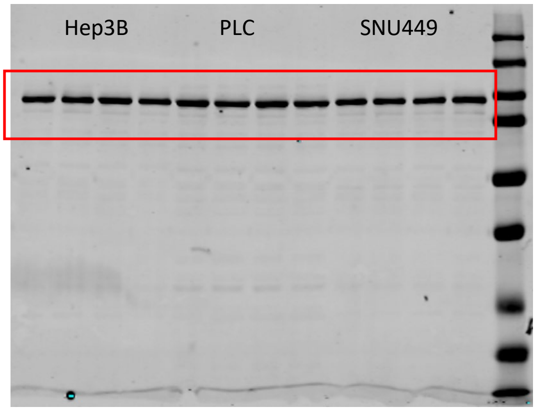

pS33/37 bcatenin (ECL)

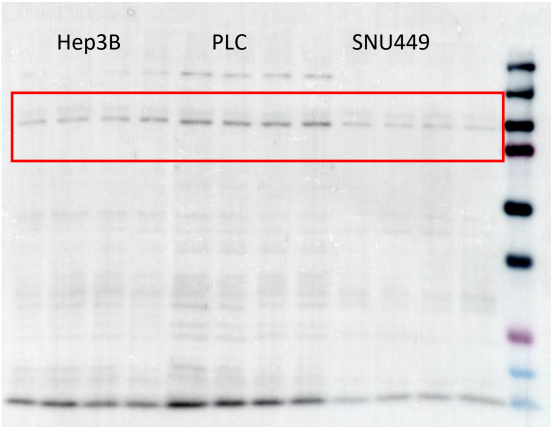

TNKS1/2 (up) and β -actin (Fluorescence)

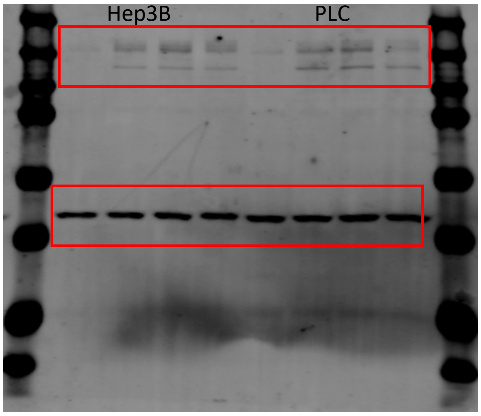

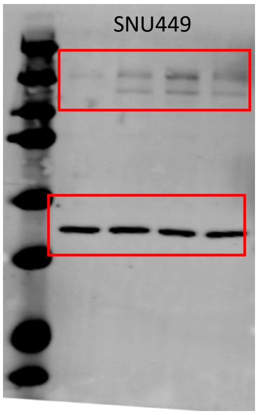

Original IB images for Figure 3: Huh7 – HepaRG – SNU182

XAV939 compound concentration : 0µM – 1µM – 5µM, IWR-1: 5µM.

N-Terminal Axin1 #3323 (ECL)

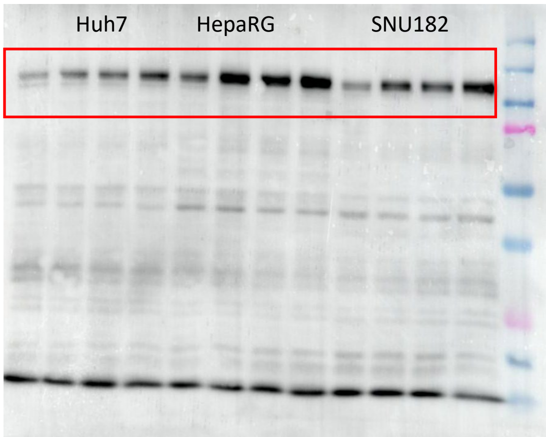

Axin2 (ECL)

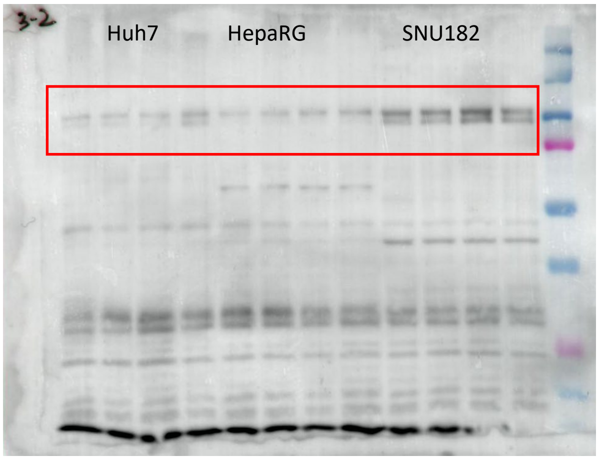

β -catenin (Fluorescence)

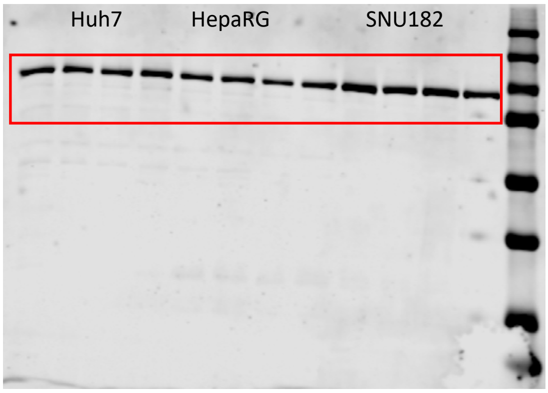

pS33/37 bcatenin (ECL)

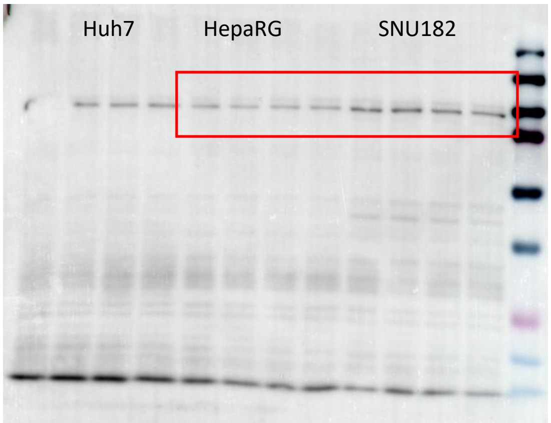

pS33/37 bcatenin (ECL)

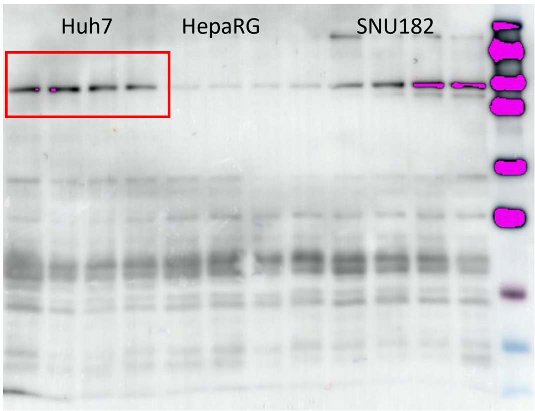

TNKS1/2 (up) and β -actin (Fluorescence)

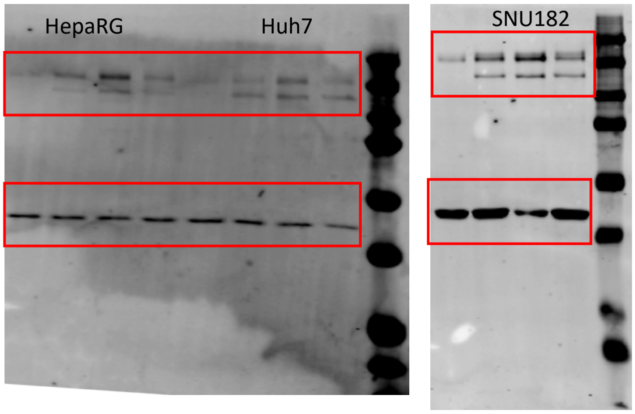

Original IB images for Figure 6: SNU449 mutant – SNU449 Repaired

XAV939 compound concentration : 0µM – 1µM – 5µM

N-Terminal Axin1 #3323 (ECL)

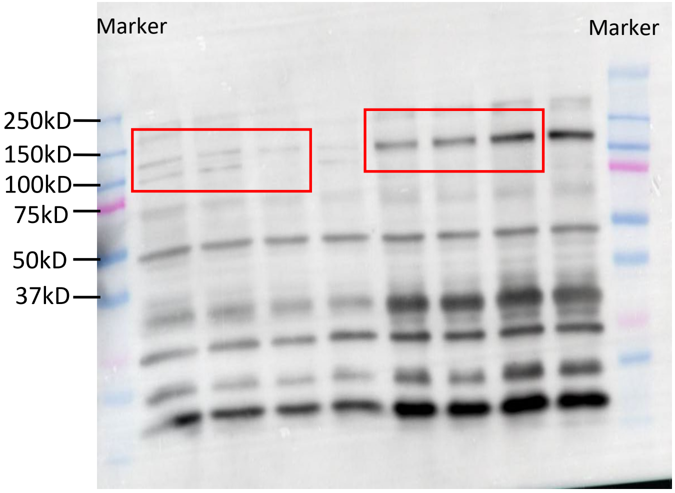

Axin 2 (ECL)

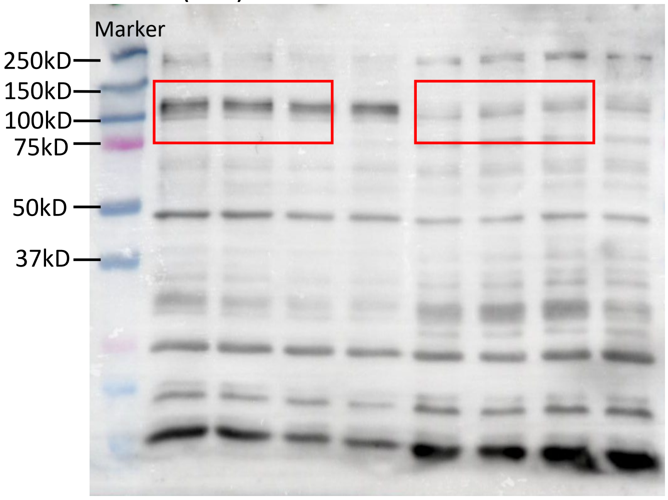

TNKS 1 / 2 and β-Actin (Fluorescence)

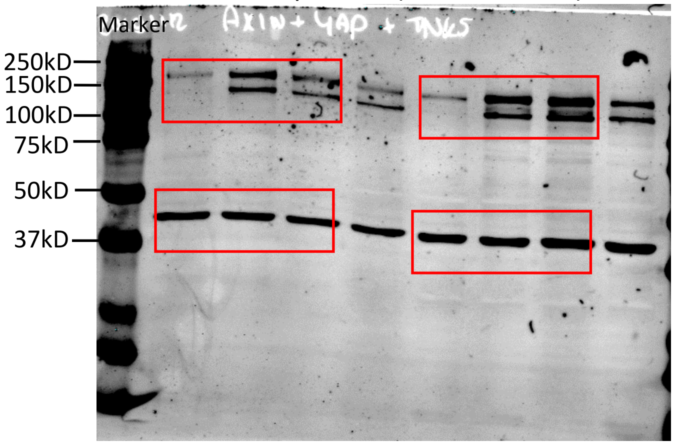

β -Catenin (Fluorescence)

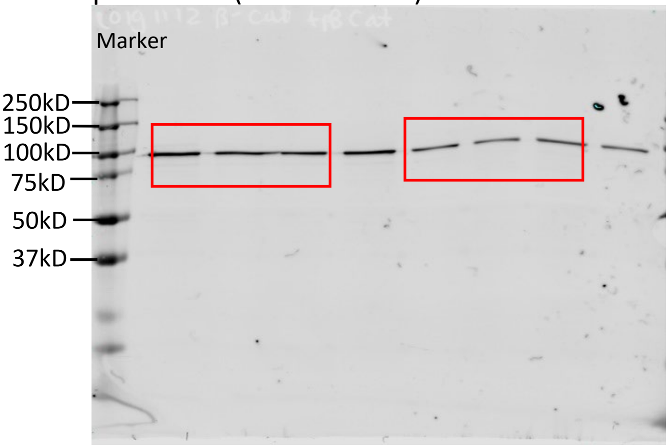

Original IB images for Figure S4:

TNKS/TNKS2 (ECL)

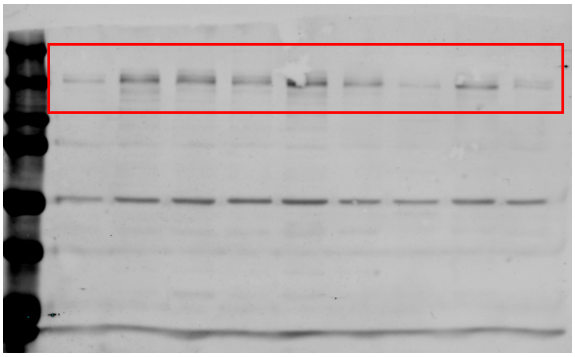

Tubulin (ECL)

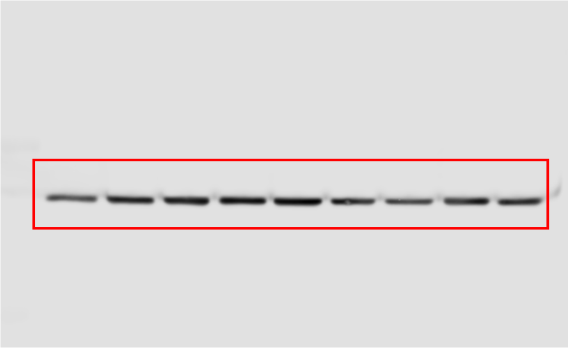

Original IB images for Figure S5: SW480 treated with XAV939 1 and 5 uM

TNKS/TNKS2 (ECL)

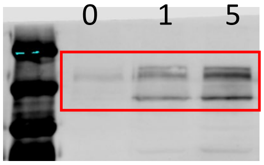

AXIN1 (ECL)

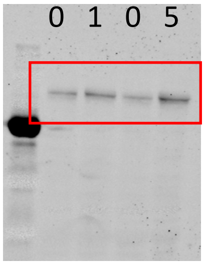

AXIN2 (ECL)

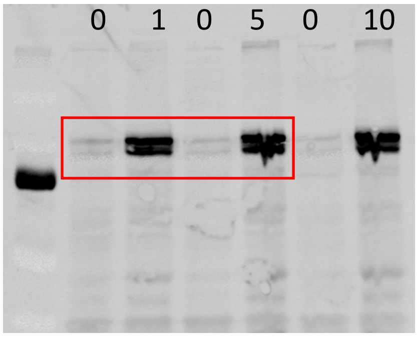

pS33/37- bcatenin (ECL)

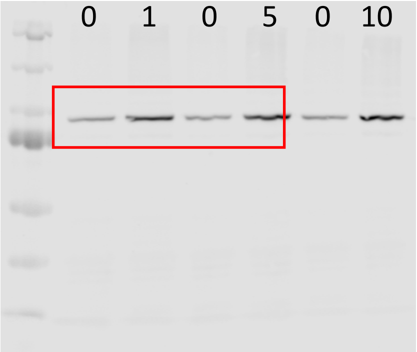

bcatenin (ECL)

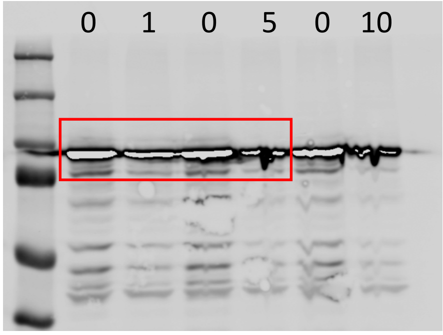

Tubulin (ECL)

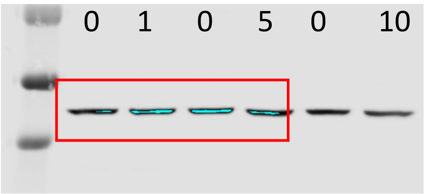

Original IB images for Figure S5: SW480 treated with IWR-1 1 and 5 uM

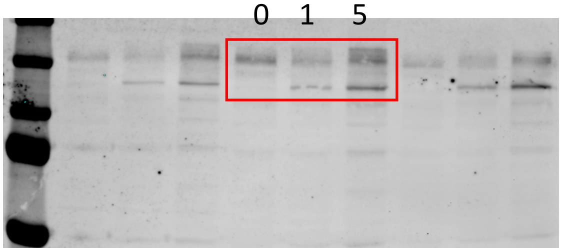

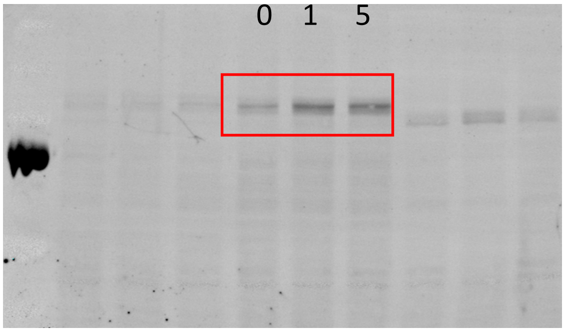

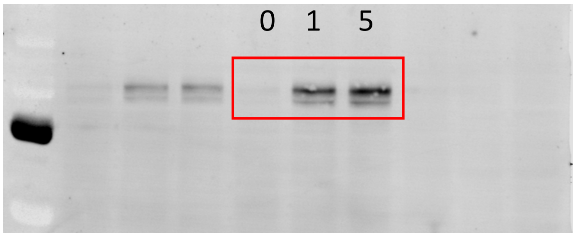

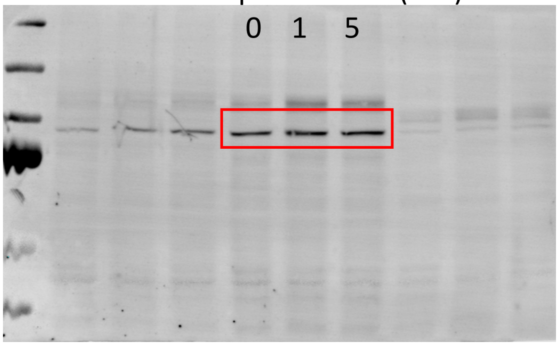

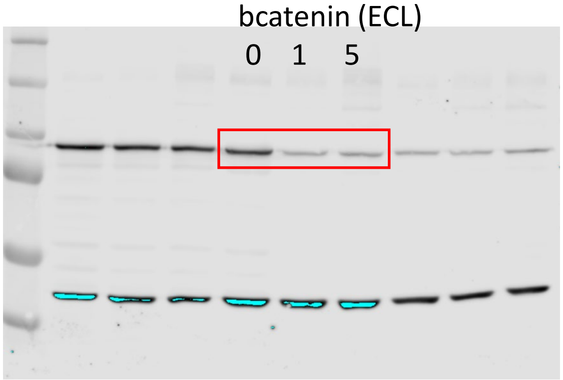

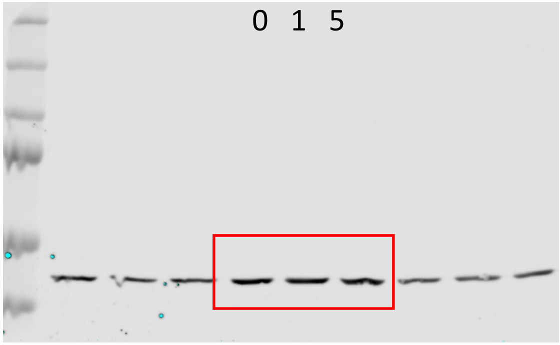

Original IB images for Figure S6: Huh7 – PLC/PRF/5 – SNU449

XAV939 compound concentration : 0µM – 1µM – 5µM – 25µM. 25µM not shown in Figure 3.

C-Terminal Axin1 #2087 (ECL)

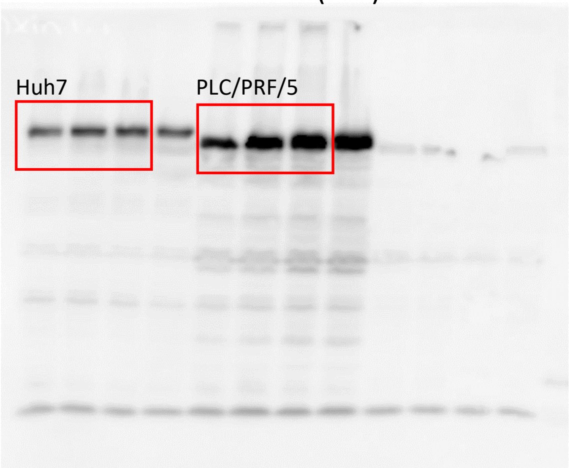

Axin2 (ECL)

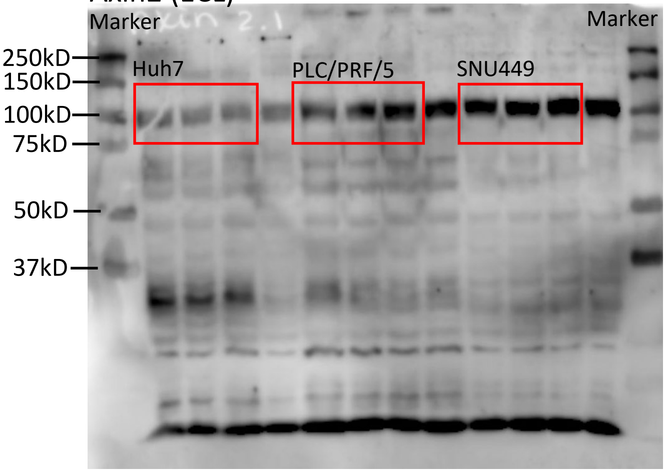

C-Terminal Axin1 #2087 (ECL)

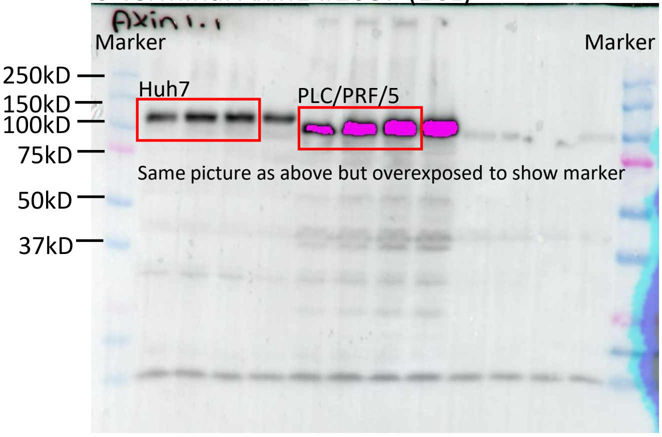

TNKS 1 / 2 (Fluorescence)

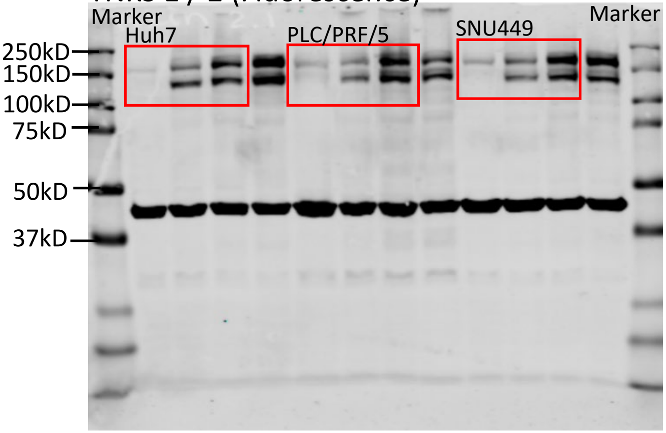

C-Terminal Axin1 #2087 (ECL)

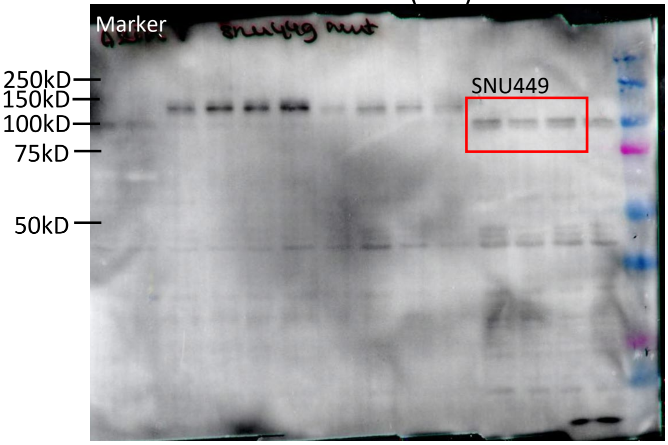

β -Actin (Fluorescence)

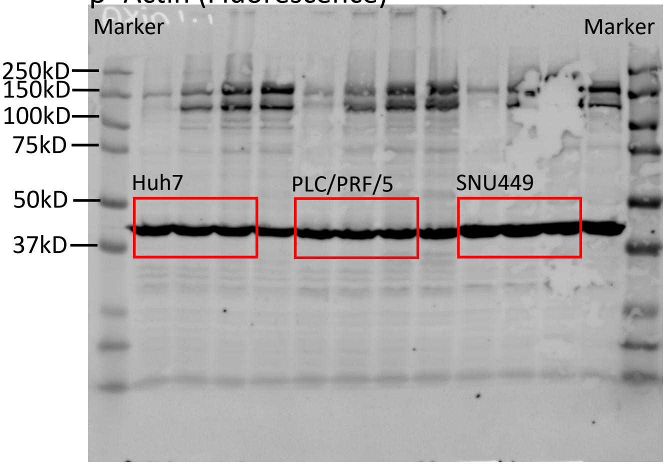

Original IB images for Figure S6: Huh7 – PLC/PRF/5 – SNU449

XAV939 compound concentration : 0µM – 1µM – 5µM.

β -Catenin (Fluorescence)

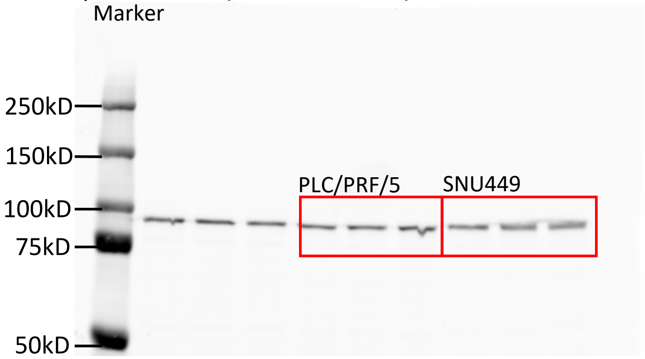

β -Catenin (Fluorescence)

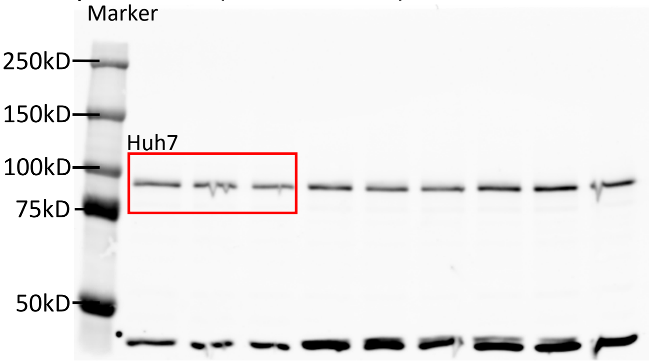

pS33/37- β -Catenin (Fluorescence)

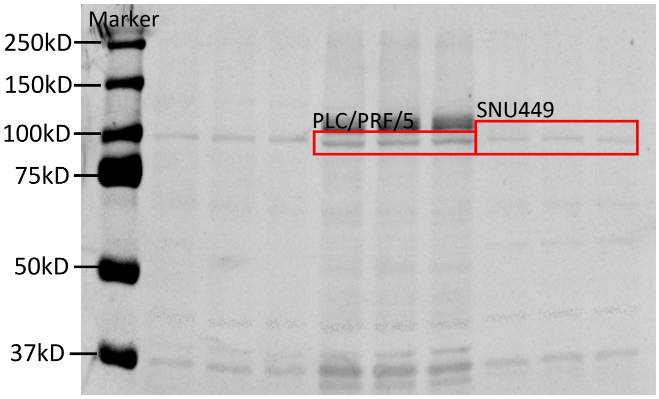

pS33/37- β -Catenin (Fluorescence)

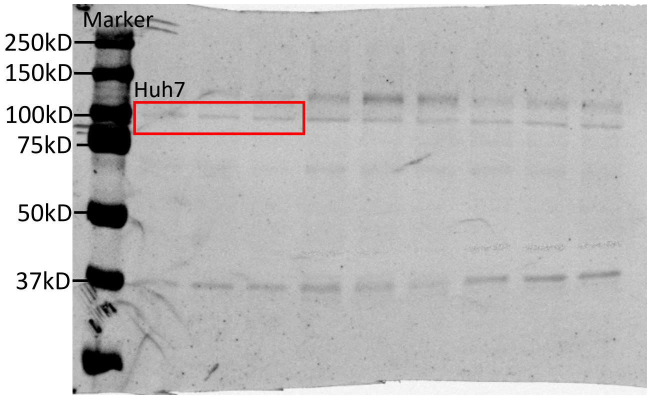

Original IB images for Figure S6: Hep3B – SNU182 - HepaRG

XAV939 compound concentration : 0µM – 1µM – 5µM – 25µM. 25µM not shown in Figure 3.

C-Terminal Axin1 #2087 (ECL)

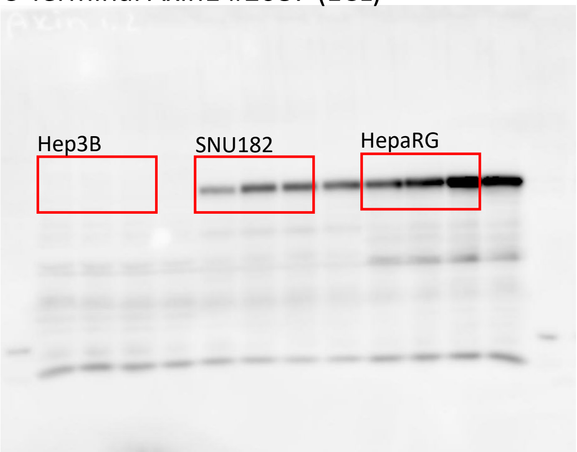

Axin2 (ECL)

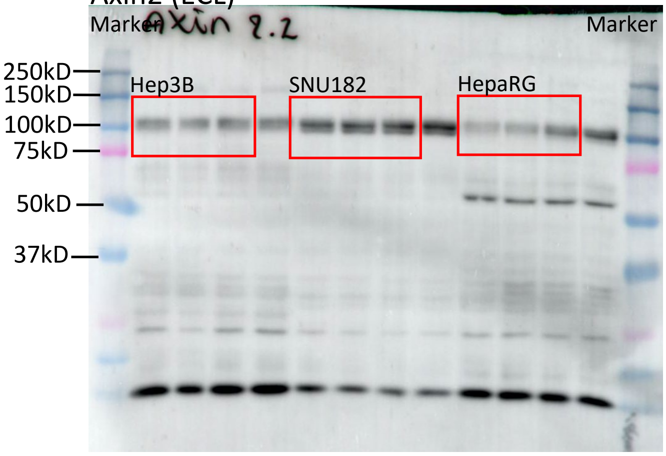

C-Terminal Axin1 #2087 (ECL)

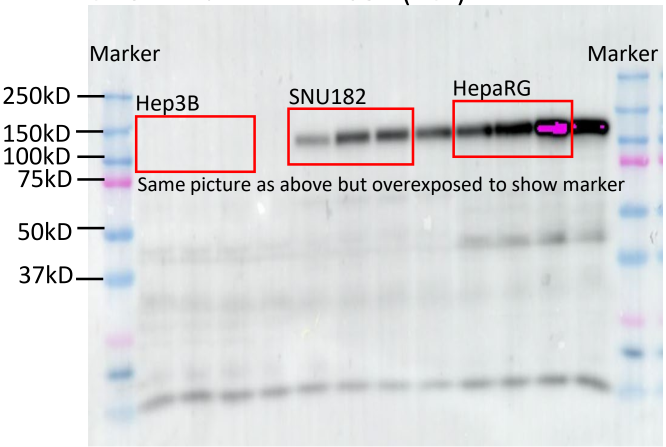

TNKS 1/2 (Fluorescence)

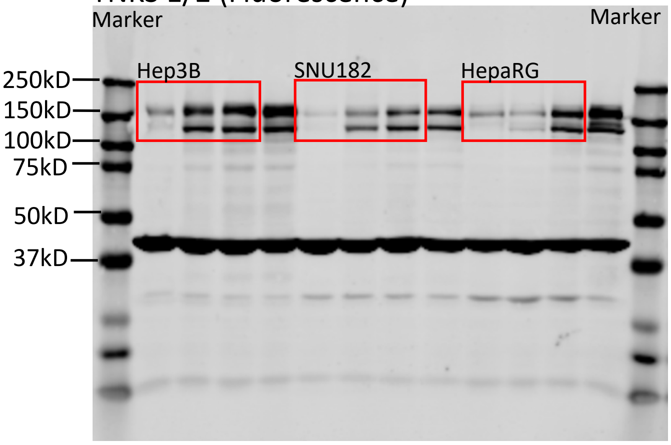

Original IB images for Figure S6: Hep3B – SNU182 - HepaRG

XAV939 compound concentration : 0µM – 1µM – 5µM.

β -Catenin (Fluorescence)

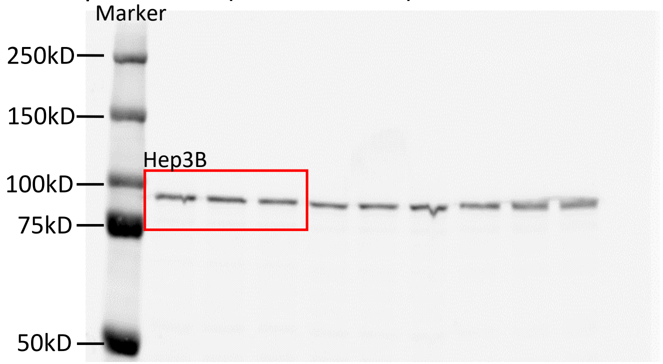

β -Catenin (Fluorescence)

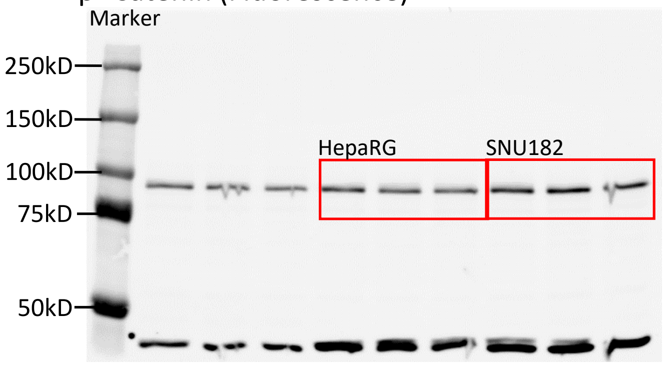

pS33/37- β -Catenin (Fluorescence)

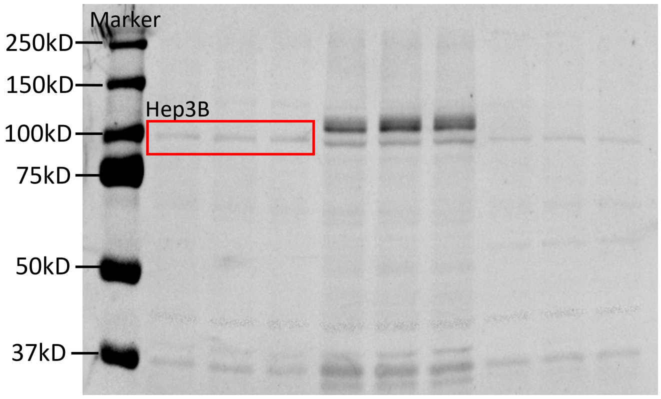

pS33/37- β -Catenin (Fluorescence)

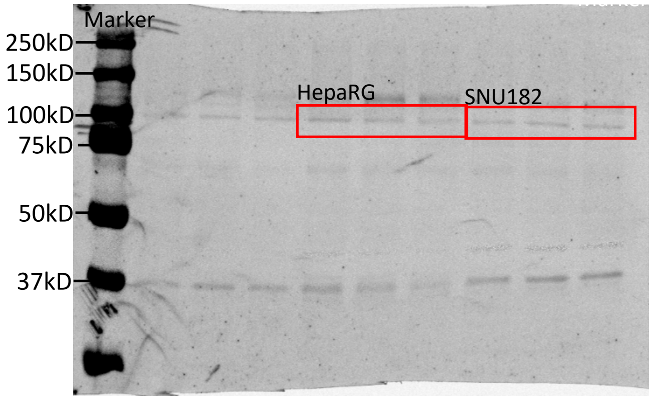

β-Actin (Fluorescence)

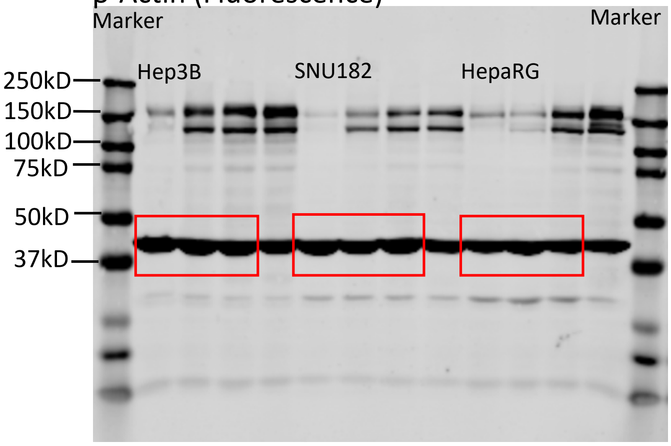

Original IB images for Figure S6: HepG2 – Huh6 – SNU398

XAV939 compound concentration : 0µM – 1µM – 5µM – 25µM. 25µM not shown in Figure 3.

C-Terminal Axin1 #2087 (ECL)

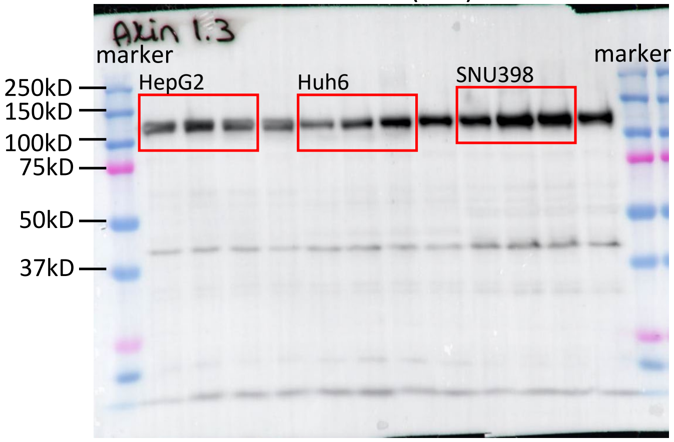

Axin2 (ECL)

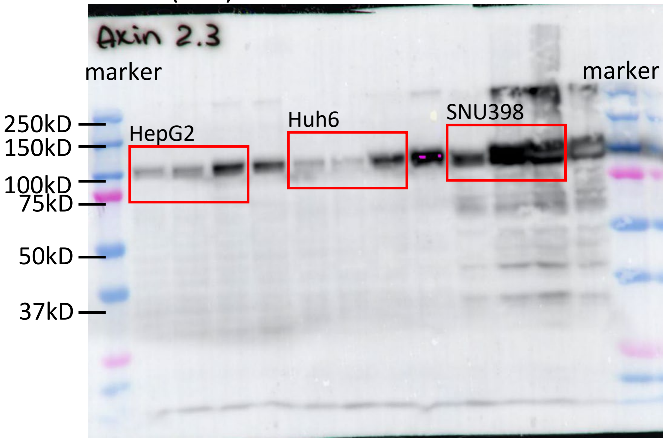

TNKS 1/2 (Fluorescence)

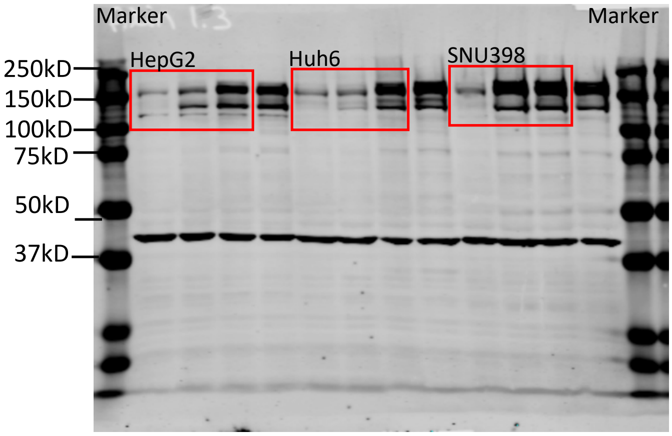

β-Actin (Fluorescence)

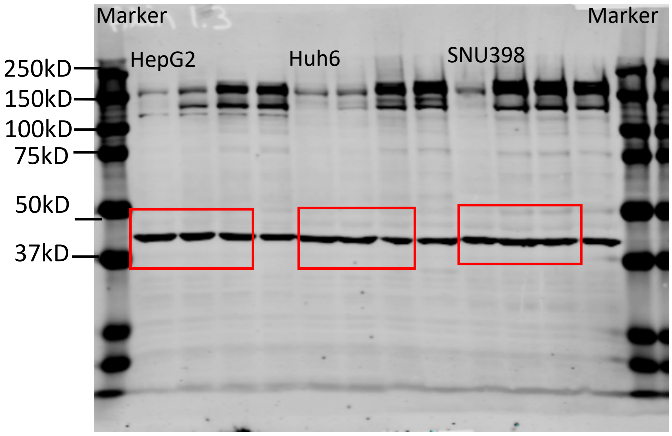

Original IB images for Figure S6: HepG2 – Huh6 – SNU398

XAV939 compound concentration : 0µM – 1µM – 5µM.

β -Catenin (Fluorescence)

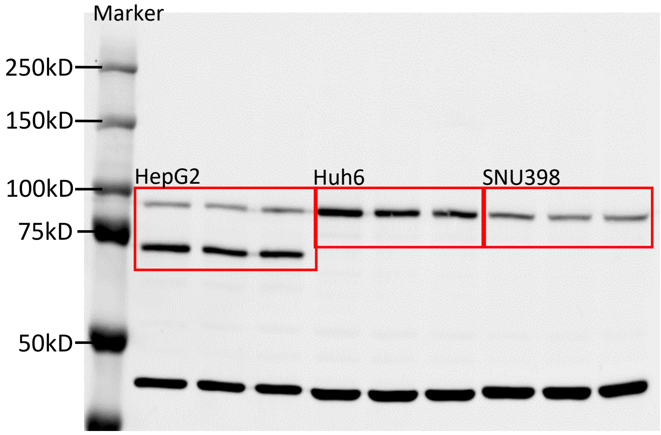

pS33/37-β-catenin (Fluorescence)

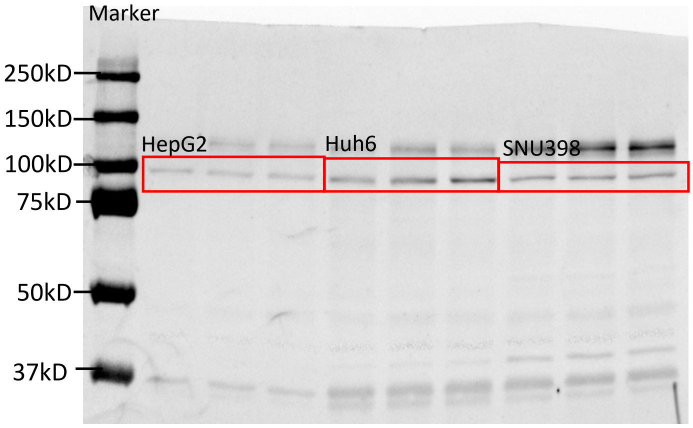

Original IB image for Figure S11: AXIN1 repaired SNU449 clones

AXIN1 (ECL)

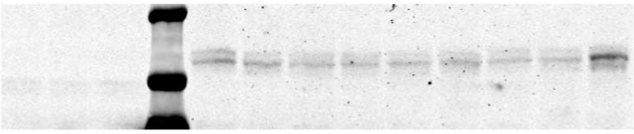

Supplement: Supplementary file 2 — Supplementary Figures. [file 41598_2021_87091_MOESM2_ESM.pdf]
